# Spatial associations among soil physicochemical properties, rhizosphere microbiota, leaf mineral status, agronomic traits, and fruit quality of white strawberry in acidic soils

**DOI:** 10.3389/fpls.2026.1927019

**Published:** 2026-09-21

**Authors:** Yan Zha, Wenfei Xiao, Shaoqiang Hu, Xiaoming Gu, Chunnan Li, Wenguo Lai, Xiaoyuan Li, Jianrong Wang, Hong Yu, Aichun Liu, Zhaoli Wang

**Affiliations:** 1 Institute of Biotechnology Research, Hangzhou Academy of Agricultural Sciences, Hangzhou, China; 2 Zhejiang Lvying Agricultural Technology Co., Ltd, Hangzhou, China; 3 Experimental Center, Hangzhou Academy of Agricultural Sciences, Hangzhou, China; 4 Horticulture Station, Agricultural Technology Extension Center of Linping District, Hangzhou, China

**Keywords:** fruit quality, leaf mineral nutrition, plant-microbe-soil interactions, rhizosphere microbiota, soil physicochemical properties

## Abstract

Soil physicochemical heterogeneity and rhizosphere microbial communities jointly influence plant nutrient acquisition and crop performance, yet their relative associations with strawberry nutritional status and productivity remain poorly understood in acidic agricultural soils. In this study, soil physicochemical properties, enzyme activities, bacterial community diversity and composition, leaf mineral status, agronomic traits, and fruit quality were systematically characterized across strawberry cultivation sites. Mantel tests, redundancy analysis (RDA), and partial least squares path modeling (PLS-PM) were used to examine the relationships among soil conditions, microbial communities, plant mineral nutrition, and strawberry performance. Pronounced spatial variation was detected in soil nutrient status, electrical conductivity (EC), bacterial community structure, leaf mineral composition, agronomic traits, and fruit-quality characteristics. Bacterial richness indices, particularly ACE and Chao1, were more strongly associated with leaf mineral accumulation than individual soil physicochemical variables or enzyme activities. In contrast, fungal Shannon and Simpson diversity indices showed stronger associations with variation in fruit quality, indicating that bacterial and fungal community attributes were differentially related to distinct components of strawberry performance. Path analysis further indicated that soil physicochemical properties were indirectly associated with plant traits primarily through bacterial α-diversity, with a particularly strong pathway linking microbial diversity to leaf mineral status. Soil-related variables accounted for a greater proportion of the variation in agronomic traits than in fruit quality. Moreover, soils enriched in different bacterial genera were associated with contrasting leaf Zn and Fe concentrations, potentially reflecting taxon-specific responses to micronutrient conditions. Spearman correlation analysis further revealed trait-specific relationships among soil fertility, mineral profiles, vegetative performance, and fruit quality, suggesting that nutrient status was differentially associated with growth and quality attributes rather than reflecting a generalized trade-off. Collectively, these results indicate complementary but distinct microbial associations: bacterial richness was more strongly associated with variation in leaf mineral profiles, whereas fungal diversity showed more specific relationships with fruit-quality traits. Rhizosphere microbial community attributes may therefore serve as potential ecological indicators of variation in strawberry nutritional status and fruit quality, rather than as experimentally validated causal drivers of plant performance.

## 1 Introduction

Strawberry (*Fragaria × ananassa* Duch.) is a perennial herbaceous fruit crop in the Rosaceae family and is widely valued for its distinctive flavor and high nutritional value. Strawberry fruit is rich in vitamin C, anthocyanins, essential minerals, and other bioactive compounds. According to statistics from the Food and Agriculture Organization of the United Nations, the annual area devoted to strawberry cultivation in China exceeds 200, 000 ha (Mekouar and Mohamed A, 2023). Total production surpassed 4 million tons in 2023, accounting for more than 40% of global strawberry production. Strawberry also has substantial economic value in global markets because of its desirable sensory characteristics and nutritional properties (Fernández-Veloso et al., 2025). In addition to its characteristic taste and aroma, strawberry fruit contains abundant phenolic compounds, anthocyanins, and vitamin C, which contribute to its antioxidant capacity (Duan et al., 2025a, 2025). These attributes have contributed to the increasing recognition of strawberry as a functional fruit with considerable nutritional and commercial value. However, strawberry production in China exhibits pronounced regional variation in both yield and fruit quality, which constrains the sustainable development of high-quality production systems.

In recent years, the cultivation of ‘Cuiyu’ has expanded in East China with a notable increase in provinces such as Zhejiang, Jiangsu, and Anhui. The performance of the plant in different field conditions has received increasing attention from researchers. Field observations have shown considerable regional variability in the growth, nutrient status of leaves, and quality of fruits in Cuiyu. This observation is consistent with previous studies showing that seasonal and edaphoclimatic variability can significantly influence the fruit quality, phytochemical composition, and biological activity of white strawberry (Vasquez et al., 2020). Plants often exhibit poor vegetative growth, leaf chlorosis due to deficiency of nutrients, and fruits with poor flavor in some production regions. Imbalances in the nutrient status of leaves remain one of the major challenges influencing the quality of strawberries produced (Osvalde et al., 2023). Consequently, understanding the causes of this regional variability is important for improving performance and fruit quality of crops.

The soil is the main substrate that promotes strawberry growth. Several studies have shown that differences in soil physicochemical properties, rhizosphere microbes, and soil enzymes largely explain geographical variations in strawberry productivity and quality, therefore emphasizing the necessity of further study on specific soil traits. Among the soil physicochemical properties of soil, the pH is considered the most important regulator. For strawberry growing, the ideal pH is 5.5-6.5; deviations from this optimal range decrease the bioavailability of micronutrients, Fe, Zn, and B, leading to nutritional deficiencies in leaves, poor photosynthesis, and fruit yield and quality (Pacheco-Ruiz et al., 2026). Soil organic matter (SOM) plays an important role as a storehouse of nutrients and improves the soil structure, its water and nutrients retention capacity. However, high soil electrical conductivity (EC) level, which refers to the presence of salts, prevents the uptake of water and nutrients by the roots and affects the fruit sugar-acid ratio negatively (Keutgen and Pawelzik, 2008). Enzyme activities in soil, which include the activities of invertase, urease, catalase, and acid phosphatase, are considered crucial for the evaluation of soil fertility and biological activity. The activity of invertase has a positive correlation with root viability and above-ground biomass (Dindar et al., 2015). The microbial communities of the rhizosphere form intimate relationships with the roots of strawberry, in which beneficial species such as *Bacillus* and *Pseudomonas*, act to inhibit pathogens and facilitate root growth, and the mycorrhizal fungi increase the root absorptive surface area and improve trace-element uptake (Balcı, 2023). Diverse microbial populations have been found to be associated with flavor compounds, and differences in the rhizosphere microbial community could affect the uptake of trace elements and the quality of fruits in white strawberries (Kwak et al., 2025). Thus, soil physicochemical properties, enzyme activities, and microbial communities form an interconnected system associated with plant mineral metabolism, vegetative growth, and fruit quality.

Although soil physicochemical properties, rhizosphere microorganisms, plant mineral nutrition, and fruit quality have been extensively studied in strawberry production systems, an important ecological question remains unresolved: how these components are hierarchically integrated within the soil–microbiome–plant continuum under acidic soil conditions. Previous studies have generally considered soil physicochemical properties, enzyme activities, and microbial communities as independent predictors, providing limited insight into whether rhizosphere microbial communities may serve as ecological links between soil heterogeneity, plant nutrient acquisition, and quality formation.

Here, we propose a hierarchical framework linking soil heterogeneity, microbial community differentiation, and plant mineral nutrition to characterize how spatial variation in soil physicochemical conditions, nutrient availability, and salinity is associated with rhizosphere microbial differentiation and, in turn, with leaf mineral status, agronomic traits, and fruit quality in white strawberry. Rather than treating microbial communities as direct causal drivers, we evaluated their potential ecological relevance as integrative indicators of variation in soil biological processes associated with nutrient transformation and plant nutrient acquisition. Specifically, we investigated whether (i) bacterial richness is more strongly associated with leaf mineral profiles than individual soil physicochemical properties or enzyme activities; (ii) bacterial and fungal communities show distinct associations with leaf mineral status, agronomic performance, and fruit quality; and (iii) relationships along the soil–microbiome–leaf mineral continuum are associated with the balance between vegetative growth and quality formation.

## 2 Materials and methods

### 2.1 Plant materials and growth conditions

Uniform plants of the white strawberry cultivar ‘Cuiyu’, bred by the Hangzhou Academy of Agricultural Sciences, were investigated at six commercial cultivation sites: Jiande Hongji Family Farm (ZS, 119.4214° E, 29.5571° N, altitude 48 m), Tonglu Xiaolu Orchard (XL, 119.5735° E, 29.9208° N, altitude 71 m), Tonglu Jianying Orchard (JY, 119.6317° E, 29.8446° N, altitude 62 m), Jiande Strawberry Garden (WS, 119.4192° E, 29.5543° N, altitude 46 m), Changfeng Orchard (CF, 117.2268° E, 32.2814° N, altitude 52 m), and the Zhijiang Base of the Hangzhou Academy of Agricultural Sciences (ZJ, 120.1281° E, 30.1395° N, altitude 16 m). These sites differed in geographic location, landform, and soil texture and therefore represented distinct commercial production environments. Because the study was conducted under field conditions, site-specific differences in environmental conditions and management practices, such as microclimate and cultivation practices, could not be completely eliminated. Cultivation site was therefore treated as a composite environmental factor integrating the measured soil physicochemical properties with potential unmeasured environmental heterogeneity. Spatial variation in soil conditions among sites was primarily associated with differences in geographic location and landform. The sampling sites encompassed several representative terrain types, including hills, plains, and river valleys, with corresponding soil textures classified as sandy loam, loam, and light clay, respectively. Collectively, the six sites represented the major soil types and field conditions under which ‘Cuiyu’ strawberry is commercially cultivated in Zhejiang and Anhui provinces.

Before transplanting, all experimental plots received the same basal fertilization according to conventional local cultivation practices, consisting of 30, 000 kg·ha^-1^ organic fertilizer and 400 kg·ha^-1^ balanced N-P_2_O_5_-K_2_O compound fertilizer (e.g., 15:15:15). In mid-to-late September, uniform, vigorous 60-day-old strawberry plants, each with four fully expanded true leaves and a height of 8–10 cm, were transplanted onto raised ridges and irrigated immediately to facilitate root establishment. Throughout the growing period, standardized greenhouse management practices, including micro-drip irrigation, temperature regulation, leaf and stolon trimming, flower and fruit thinning, and integrated pest management (IPM), were applied uniformly across all sites to minimize management-related variation. During flowering and fruiting, a standardized fertigation regime using water-soluble fertilizers was applied periodically through the drip-irrigation system. Fruits were harvested in early January of the following year upon reaching commercial ripeness, as indicated by a milky-white peel with a slight pink blush. Paired soil, leaf, and fruit samples were collected synchronously from each of the six cultivation sites for subsequent analyses of nutrient concentrations and fruit quality.

### 2.2 Soil and strawberry sample collection

At each cultivation site, three separate commercial greenhouses were selected as independent experimental units. Each greenhouse covered approximately 400 m^2^ (8 m × 50 m), and adjacent greenhouse units were separated by at least 5 m. Within each site, the selected greenhouses were comparable in crop age, planting density, cultivation history, and management practices, thereby minimizing within-site environmental variation. In total, 18 greenhouses were included in the study, and spatial variation was evaluated among greenhouse-level experimental units across the six cultivation sites.

Within each greenhouse, a central 20 m^2^ sampling area comprising two adjacent 10-m-long raised beds was established. To minimize edge effects, the sampling area was positioned at least 5 m from the greenhouse entrance and end walls and at least 1 m from the sidewalls. Soil, leaf, and fruit subsamples collected within each greenhouse were subsequently pooled or averaged to obtain greenhouse-level measurements for statistical analysis.

Immediately after homogenization, each composite bulk-soil sample was divided into three portions. The first portion was air-dried at room temperature and passed through a 2-mm sieve for analysis of soil physicochemical properties. The second portion was maintained in a field-moist state, passed through a 2-mm sieve to remove visible roots, stones, and plant residues, and used for soil enzyme activity assays within 24 h of sampling. Before enzyme analysis, these fresh soil samples were stored at 4 °C; air-dried soil was not used for enzyme activity measurements. The third portion was used to determine soil moisture content, allowing enzyme activities to be expressed on an oven-dry soil-equivalent basis.

Rhizosphere soil was collected from five randomly selected healthy plants in each greenhouse. After loosely adhering soil had been removed, soil tightly adhering to the roots was collected using sterile instruments. Equal amounts of rhizosphere soil from the five plants were combined to form one composite rhizosphere-soil sample per greenhouse. Samples were immediately transported on dry ice and stored at -80 °C until DNA extraction. Accordingly, each greenhouse yielded one composite bulk-soil sample, one composite rhizosphere-soil sample, pooled leaf samples, and pooled fruit samples. The final dataset therefore comprised 18 greenhouse-level biological replicates distributed across the six cultivation sites. The greenhouse was considered the experimental unit, whereas individual plants and soil cores were treated as subsamples rather than independent biological replicates.

### 2.3 Soil physical and chemical property determination

Soil physicochemical properties were determined according to the methods described in Methods of Soil Agricultural Chemical Analysis (Lu, 2000). Soil pH was measured potentiometrically at a soil-to-water ratio of 1:2.5. Electrical conductivity (EC) was measured using a standard conductivity meter. Soil organic matter (SOM) was determined using the potassium dichromate oxidation-external heating method. Alkaline hydrolyzable nitrogen (AN) was quantified using the alkaline hydrolysis diffusion method. Available phosphorus (AP) was determined using the molybdenum-antimony colorimetric method, whereas available potassium (RAP) was measured using a flame photometer. Exchangeable calcium (Ca) and magnesium (Mg), together with available iron (Fe), manganese (Mn), and zinc (Zn), were determined by atomic absorption spectrophotometry.Detailed analytical procedures for the determination of soil physicochemical properties are provided in **Supplementary Methods S1**.

### 2.4 Soil enzyme activities

Fresh, field-moist soil samples were passed through a 2-mm sieve to remove visible roots, stones, and plant residues and were used for enzyme activity assays immediately after sampling. Before analysis, the samples were stored at 4 °C without air-drying or grinding. Soil sucrase (SC) activity was determined using the 3, 5-dinitrosalicylic acid (DNS) colorimetric method under acidic buffer conditions, as described by Miller (1959). Soil urease (Urease) activity was quantified using the phenol-sodium hypochlorite colorimetric method (Tabatabai and Bremner, 1969), whereas acid phosphatase (ACPT) activity was measured using the disodium phenyl phosphate colorimetric method (Tabatabai and Bremner, 1972). Soil acid protease (ACP) activity was determined using the Folin–phenol colorimetric method with casein as the reaction substrate. For each enzyme assay, substrate concentration, buffer system, reaction temperature, and incubation time were kept consistent across all samples. Each biological sample was analyzed in triplicate. Enzyme activities were corrected for soil moisture content and expressed on an oven-dry soil-equivalent basis. Detailed analytical procedures for the determination of soil enzyme activities are provided in **Supplementary Methods S2**.

### 2.5 Microbial DNA extraction, amplicon sequencing, and bioinformatic analysis

Rhizosphere soil samples were collected from 18 experimental units, with three biological replicates for each treatment. Total microbial DNA was extracted from 0.50 g of homogenized rhizosphere soil using the CretMag™ Power Soil DNA Kit (China) according to the manufacturer’s instructions. DNA integrity was assessed by 1% agarose gel electrophoresis, and DNA concentration and purity were determined using a NanoDrop 2000 spectrophotometer.

The V3-V4 region of the bacterial 16S rRNA gene was amplified using primers 341F (5′-CCTAYGGGRBGCASCAG-3′) and 806R (5′-GGACTACHVGGGTWTCTAAT-3′), whereas the fungal ITS region was amplified using primers ITS1F (5′-CTTGGTCATTTAGAGGAAGTAA-3′) and ITS2R (5′-GCTGCGTTCTTCATCGATGC-3′). PCR amplification was performed using 2× ES Taq MasterMix (Dye) under the following thermal cycling conditions: initial denaturation at 94 °C for 2 min; 30 cycles of denaturation at 94 °C for 30 s, annealing at 55 °C for 30 s, and extension at 72 °C for 30 s; followed by a final extension at 72 °C for 10 min. Triplicate PCR products from each sample were pooled, purified, and sequenced on the DNBSEQ-G99 PE300 platform (BGI, China).

Following paired-end read merging and quality filtering, 1, 924, 519 bacterial 16S rRNA gene reads and 3, 396, 833 fungal ITS reads were obtained from 18 rhizosphere soil samples. The number of high-quality bacterial tags retained per sample ranged from 57, 580 to 120, 402, with a mean of 82, 866. The number of high-quality fungal tags ranged from 145, 012 to 251, 271 per sample, with a mean of 179, 457 (**Supplementary Tables S1**, **S2**). Raw paired-end reads were quality-filtered using Trimmomatic (v0.36), merged using FLASH (v1.20) and PEAR (v0.9.6), and screened for chimeric sequences using UCHIME implemented in VSEARCH (v2.7.1). The quality-filtering criteria included a 50-bp sliding window, a minimum mean quality score of Q20, a minimum sequence length of 120 bp, a minimum overlap of 10 bp, and a maximum mismatch rate of 0.1.

Sequence analyses were performed using QIIME2 (v2023.9) and VSEARCH (v2.7.1). Clean sequences were clustered into operational taxonomic units (OTUs) at 97% sequence similarity using the UPARSE strategy. Using this OTU-based approach rather than amplicon sequence variant (ASV) inference, 1, 383-2, 774 bacterial OTUs and 394–659 fungal OTUs were detected per sample. Taxonomic assignment was performed using the SILVA database (release 132) for bacterial sequences and the UNITE database (release 7.2) for fungal sequences.

Before α-diversity analysis, the OTU tables were rarefied to the minimum sequencing depth of each dataset by subsampling without replacement, following Weiss et al. (2017), to reduce bias caused by unequal library sizes. Specifically, the bacterial and fungal OTU tables were rarefied to 57, 795 and 145, 012 tags per sample, respectively. The rarefied matrices were used exclusively to calculate α-diversity, whereas β-diversity analyses were performed using the unrarefied OTU tables to avoid unnecessary loss of sequencing information. Sequencing depth was considered sufficient based on rarefaction curves and Good’s coverage values exceeding 99%. Detailed analytical procedures for the determination of rhizosphere microbiota are provided in **Supplementary Methods S3**.

The sequencing data supporting this study have been deposited in the China National GeneBank Sequence Archive (CNSA, https://db.cngb.org/cnsa/) under accession number CNP0009969 (Wang et al., 2025).

### 2.6 Strawberry phenotypic and nutrients indicators

Six representative strawberry plants were randomly selected from each greenhouse and treated as subsamples rather than as independent biological replicates. Trait measurements from these plants were averaged to obtain one greenhouse-level value for inferential statistical analysis. Thus, each greenhouse was considered an independent experiment unit, with three independent greenhouses per cultivation site and 18 greenhouse-level observations in total. For each treatment, six plants were randomly selected as subsamples for the determination of strawberry agronomic traits.

Plant height was measured using a graduated ruler, whereas stem and crown diameters were measured using a digital vernier caliper. Leaf area was determined using a leaf area meter. To determine plant biomass, aboveground and belowground tissues were carefully separated, and surface debris and other impurities were removed with deionized water. The fresh weight of each tissue was measured immediately using a high-precision analytical balance. All plant samples were subsequently oven-dried at 65 °C to a constant weight for dry-biomass determination. Major mineral elements in leaves were quantified using standard analytical procedures. Leaf total nitrogen (N) was measured after sulfuric acid-hydrogen peroxide digestion using a continuous-flow analyzer (Umar et al., 2023), consistent with the classical Kjeldahl digestion principle (Sáez-Plaza et al., 2013). Total phosphorus (P) was measured using the molybdenum-antimony colorimetric method (Murphy and Riley, 1962), and total potassium (K) was determined by flame photometry. The concentrations of calcium (Ca), magnesium (Mg), and trace elements, including iron (Fe), manganese (Mn), and zinc (Zn), were quantified using atomic absorption spectrophotometry (Lu, 2000). Detailed analytical procedures for the determination of strawberry nutrient indicators are provided in **Supplementary Methods S4**.

### 2.7 Strawberry fruit quality indicators

Strawberry fruit quality indices were determined following Wang et al. (2023), with minor modifications for the present study. Individual fruit weight was measured using a high-precision electronic balance. Fruit firmness was determined using a digital texture analyzer under standardized testing conditions. The total soluble solid (TSS) content of the fruit pulp was measured at room temperature using a handheld refractometer. Soluble sugar content was quantified using the phenol–sulfuric acid colorimetric method, and vitamin C (Vc) concentration was determined by conventional iodometric titration. Titratable acidity (TA) was measured by standard NaOH titration and expressed as citric acid equivalents in accordance with standard procedures for evaluating strawberry fruit quality. Detailed analytical procedures for the determination of strawberry fruit quality indicators are provided in **Supplementary Methods S5**.

### 2.8 Statistical analysis

All statistical analyses were conducted in R v4.3.1 unless otherwise specified. Each greenhouse was treated as an independent experimental unit, resulting in 18 greenhouse-level observations across six cultivation sites. The six strawberry plants sampled within each greenhouse were considered subsamples, and plant-level measurements were averaged to obtain one greenhouse-level value before inferential statistical analysis. Quantitative data are presented as mean ± SD. Normality and homogeneity of variance were assessed using the Shapiro–Wilk and Levene tests, respectively (Shapiro and Wilk, 1965; Levene, 1960). Differences among cultivation sites were evaluated using one-way analysis of variance (ANOVA) followed by Duncan’s multiple-range test when parametric assumptions were satisfied; otherwise, the Kruskal–Wallis test followed by Dunn’s *post hoc* pairwise comparisons was applied (Dunn, 1964). Where appropriate, P values were adjusted using the Benjamini–Hochberg false discovery rate procedure (Benjamini and Hochberg, 1995), and statistical significance was defined as P < 0.05.

The sequencing quality and OTU statistics of bacterial 16S rRNA and fungal ITS amplicons for rhizosphere soil samples are summarized in **Supplementary Tables S1**, **S2**, respectively. Microbial α-diversity indices were analyzed using the same statistical procedures. Differences in bacterial and fungal community composition were evaluated using permutational multivariate analysis of variance (PERMANOVA) based on Bray–Curtis dissimilarities with 999 permutations after verifying the homogeneity of multivariate dispersion (Anderson, 2001). Differentially abundant microbial taxa were identified using linear discriminant analysis effect size (LEfSe) with an LDA score threshold of 2.0 (Segata et al., 2011).

Spearman rank correlations were used to evaluate pairwise associations among soil physicochemical properties, enzyme activities, microbial α-diversity, leaf mineral concentrations, agronomic traits, and fruit-quality variables. Mantel tests with 9, 999 permutations were performed to evaluate matrix-level associations using Euclidean distance matrices for standardized environmental and plant variables and Bray–Curtis dissimilarity matrices for microbial community composition. Redundancy analysis (RDA) was conducted using Canoco 5.0 to quantify the associations of soil and microbial predictors with leaf mineral profiles (TerBraak and Šmilauer, 2002). Explanatory variables were standardized before analysis, and collinearity was assessed using variance inflation factors (VIFs) and pairwise correlation coefficients. Variables with VIF ≥ 10 or pairwise correlation coefficients > 0.80 were excluded during initial screening. Statistical significance of constrained axes and individual predictors was evaluated using Monte Carlo permutation tests with 999 permutations.

Partial least squares path modeling (PLS-PM) was implemented using the R plspm package (Sanchez and Trinchera, 2012) to explore direct, indirect, and total associations among soil physicochemical properties, enzyme activities, microbial community attributes, leaf mineral status, agronomic traits, and fruit quality. The structural model was specified *a priori* according to the proposed soil–microbe–plant framework, and continuous variables were standardized using z-score transformation. Potential outliers were screened using standardized values (|Z| > 3) and checked against the original experimental records. Model complexity was constrained by retaining only theoretically justified latent constructs and removing redundant indicators during measurement-model evaluation. Indicators with absolute outer loadings below 0.40 were removed, whereas indicators with loadings of 0.40–0.70 were retained only when theoretically justified and when their inclusion did not compromise composite reliability or average variance extracted (AVE). Model robustness was evaluated using bootstrap resampling with 5, 000 iterations and leave-one-site-out sensitivity analysis. Model performance was summarized using R² and goodness-of-fit (GoF). Because this study was observational and involved a limited number of independent greenhouse-level observations, PLS-PM was used as an exploratory tool to identify patterns of covariation rather than to establish causal relationships.

## 3 Results

### 3.1 Spatial variation of soil physicochemical characteristics

As shown in **Table 1**, JY had the highest SOM, HN, CEC, and EC among all sites. The SOM, AHN, CEC, and EC values at JY were 1.97, 2.19, 3.61, and 1.56 times the corresponding values at WS, respectively. Regarding available mineral nutrients, CF had the highest (AP) concentration (155.20 mg·kg^-1^), which was approximately 12.03 times that at WS, where the lowest AP concentration was recorded. HS had the highest RAP concentration (332.33 mg·kg^-1^), approximately 11.30 times that at WS, whereas XL had an intermediate RAP concentration of 244.67 mg·kg^-1^. Soil pH ranged from 4.41 at ZS to 5.54 at HS, indicating that all soils in the study area were acidic. EC varied markedly from 289.43 μS·cm^-1^ at WS to 924.25 μS·cm^-1^ at JY, representing a 3.19-fold difference between the two extremes. The wider variation in EC than in soil pH indicated greater spatial heterogeneity in soil salinity than in soil acidity across the study region.

**Table 1:** Soil physicochemical properties at the different sampling sites.

Sampling site	SOM (g·kg^-^¹)	HN(mg·kg^-^¹)	AP(mg·kg^-^¹)	RAP (mg·kg^-^¹)	CEC(cmol·kg^-^¹)	pH	EC(μS·cm^-^¹)
ZS	20.20 ± 2.04b	100.63 ± 3.10a	96.87 ± 4.43b	99.73 ± 2.84d	15.97 ± 0.15b	4.41 ± 0.04d	688.10 ± 16.07b
WS	14.97 ± 1.56c	72.77 ± 2.41b	12.90 ± 2.08e	29.17 ± 2.12e	15.03 ± 0.67b	4.72 ± 0.08c	289.43 ± 3.91f
CF	17.67 ± 1.42b	100.63 ± 3.10a	155.20 ± 4.73a	135.00 ± 4.36c	13.10 ± 0.42c	4.71 ± 0.12c	520.37 ± 2.95e
XL	14.96 ± 1.56c	63.37 ± 10.48c	60.23 ± 4.20e	244.67 ± 5.39b	8.20 ± 0.61e	5.26 ± 0.08b	591.17 ± 4.86c
JY	27.60 ± 0.10a	139.33 ± 5.51a	78.27 ± 2.39	228.67 ± 11.15b	29.57 ± 2.00a	5.34 ± 0.08b	924.25 ± 6.34a
HS	18.37 ± 1.33b	76.13 ± 1.53b	108.8 ± 0.28d	332.33 ± 31.39a	10.83 ± 0.05d	5.54 ± 0.11a	549.53 ± 11.79d

Values are presented as means ± SD (n = 3). Different superscript lowercase letters within the same column indicate significant differences among sampling sites according to ANOVA followed by Duncan’s multiple range test (P < 0.05). No. plant^-^¹, number per plant; ZS, Jiande Hongji Family Farm; WS, Jiande Strawberry Garden; CF, Changfeng Orchard; XL, Tonglu Xiaolu Orchard; JY, Tonglu Jianying Orchard; HS, Hangzhou Academy of Agricultural Sciences. SOM, Soil organic matter; HN, Hydrolyzable nitrogen; AP, Available phosphorus; RAP, Available potassium; CEC, Cation exchange capacity; EC, Electrical conductivity.

### 3.2 Spatial variation in soil enzyme activities

One-way ANOVA revealed significant differences in the activities of all four soil enzymes among the six sampling sites (**Figure 1**). Acid protease activity was highest at WS (78.73 μg·h^-1^·g^-1^), 5.72-fold higher than that at JY. JY and XL had the lowest and comparable acid protease activities. Overall, acid protease activity followed the order WS > ZS > HS > CF > JY = XL. Urease activity was highest at ZS (756.22 μg·d^-1^·g^-1^) and lowest at JY (270.73 μg·d^-1^·g^-1^), representing a 1.79-fold difference. Urease activity differed significantly among all sites and followed the order ZS > HS > CF > WS > XL > JY. Sucrase activity ranged from 3.27 mg·d^-1^·g^-1^ at XL to 27.08 mg·d^-1^·g^-1^ at ZS, corresponding to a 7.29-fold variation between the two extremes. The overall gradient in sucrase activity was ZS > CF > WS > HS > JY > XL. Acid phosphatase activity was highest at CF (2, 891.49 nmol·h^-1^·g^-1^) and lowest at XL (331.96 nmol·h^-1^·g^-1^), representing a 7.71-fold difference among the sampling sites. Acid phosphatase activity followed the order CF > ZS > JY > WS = HS > XL.

**Figure 1:**
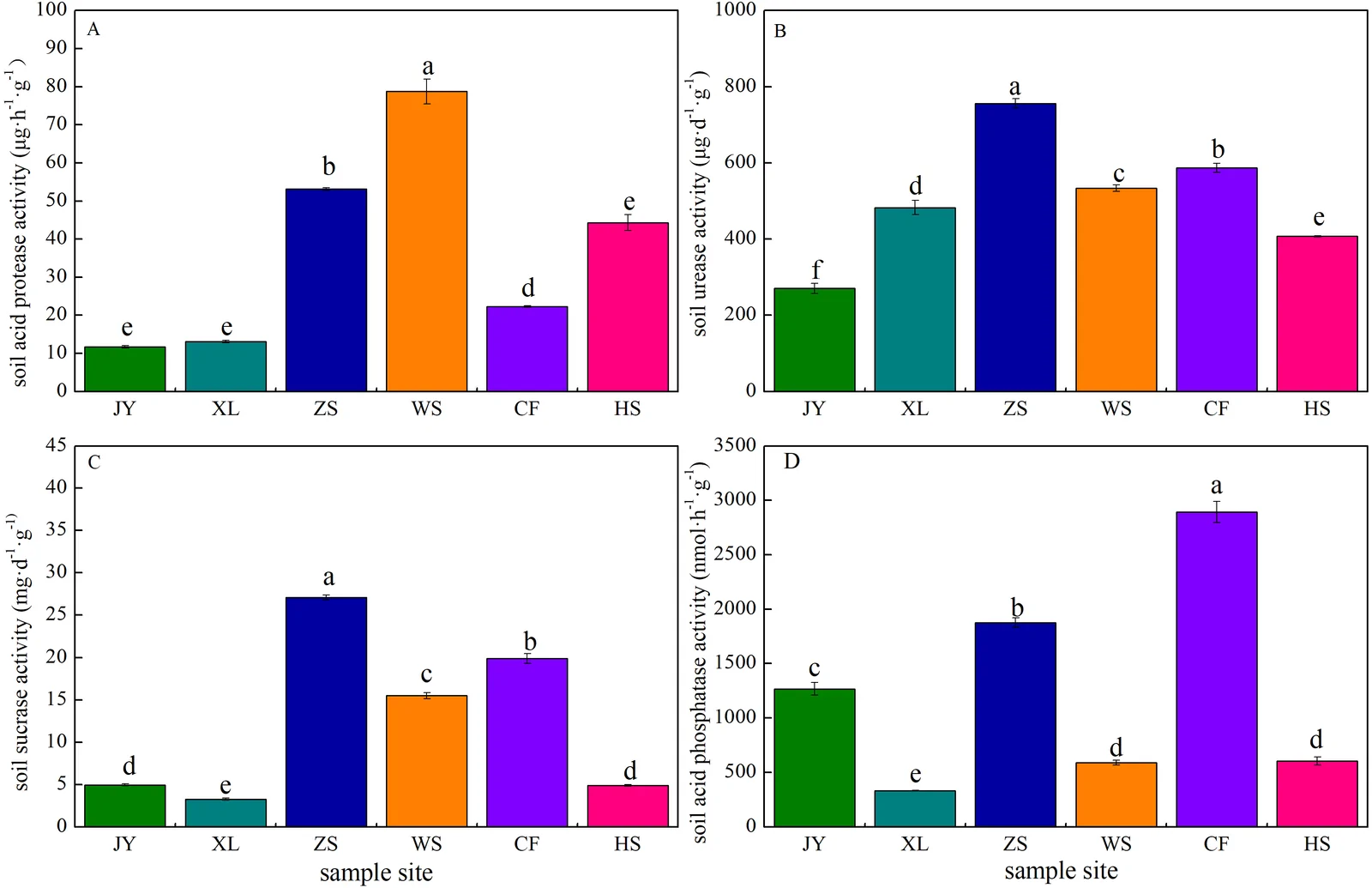
Soil enzyme activities across different sampling sites. **(A)** Soil acid protease activity. **(B)** Soil urease activity. **(C)** Soil sucrase activity. **(D)** Soil acid phosphatase activity. Values are presented as means ± SD (n = 3). Different lowercase letters above the bars indicate significant differences among sampling sites, as determined by ANOVA followed by Duncan’s multiple-range test (P < 0.05). JY, Tonglu Jianying Orchard; XL, Tonglu Xiaolu Orchard; ZS, Jiande Hongji Family Farm; WS, Jiande Strawberry Garden; CF, Changfeng Orchard; HS, Hangzhou Academy of Agricultural Sciences. ACPT, acid phosphatase; SC, soil sucrase; ACP, soil acid protease; Urease, soil urease.

### 3.3 Spatial variation in soil microbial community diversity and composition

#### 3.3.1 Spatial variation in soil microbial α-diversity

Soil bacterial richness was evaluated using the Chao1 and ACE indices. As shown in **Figure 2**, bacterial richness followed the order JY > ZS > WS > HS > CF > XL. JY had the highest Chao1 and ACE values, which were 75.27% and 73.37% higher, respectively, than those at XL (P < 0.01). ZS also exhibited significantly greater richness than XL (P < 0.01), whereas XL had the lowest bacterial richness among all sites (**Figure 2A**). The bacterial Shannon and Simpson indices showed a similar pattern (**Figure 2B**). XL had the lowest Shannon index, which was 33.6% lower than that at JY. Its Simpson index was 12.14%-13.23% lower than those at the other sites, indicating a less even community distribution. By contrast, JY and CF had more stable bacterial communities. Fungal Chao1 and ACE indices showed highly significant intersite differences and followed the order JY > ZS > CF > XL > WS > HS. Fungal richness at JY was 49.86% higher based on Chao1 and 50.08% higher based on ACE than that at HS (P < 0.01), where the lowest fungal richness was observed. No significant differences were detected in the fungal Shannon or Simpson indices (P > 0.05). Fungal communities at JY, ZS, and CF exhibited greater evenness, whereas WS showed a relatively uneven distribution.

**Figure 2:**
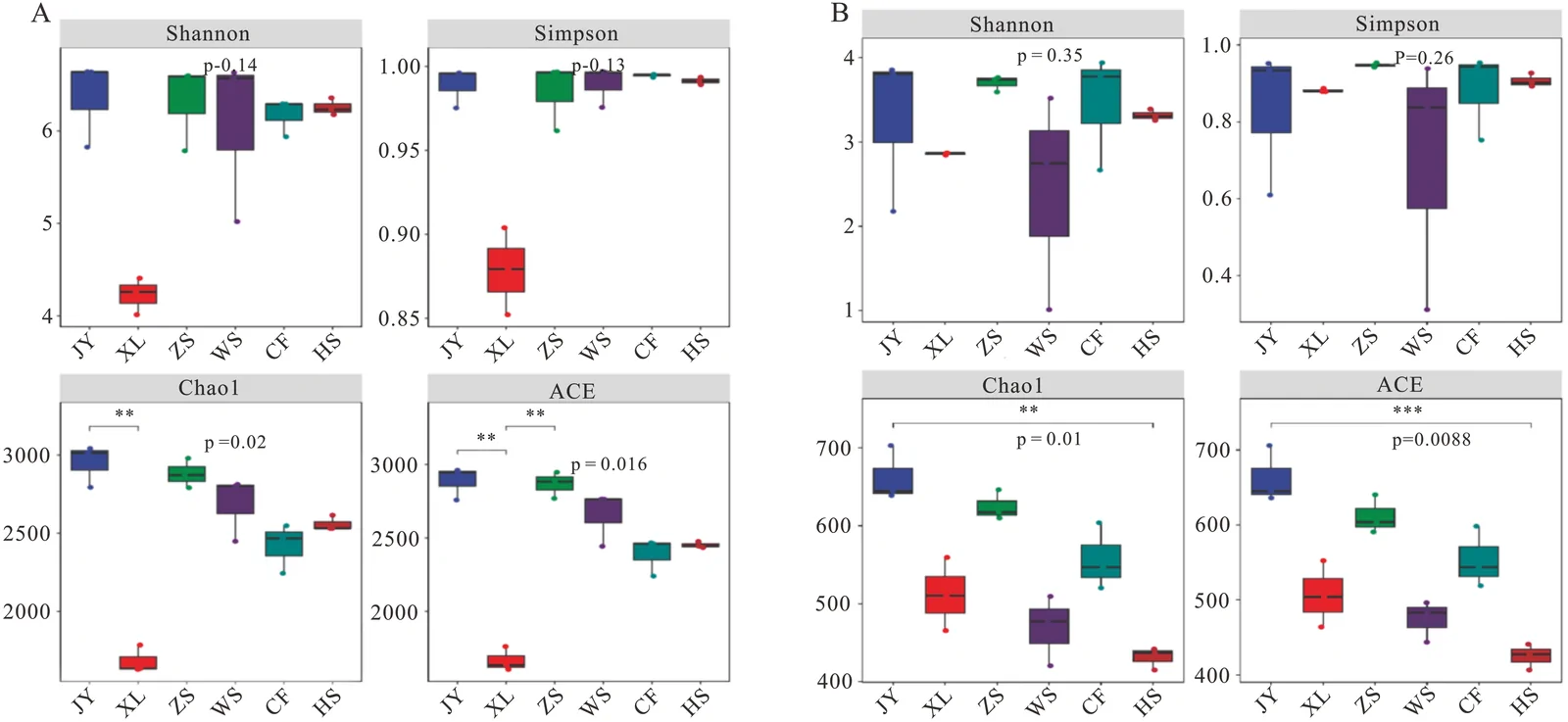
Alpha diversity of rhizosphere microbial communities across six white strawberry cultivation sites. Alpha-diversity indices of **(A)** bacterial and **(B)** fungal communities, including the Shannon, Simpson, Chao1, and abundance-based coverage estimator (ACE) indices. Each site comprised three biological replicates (n = 3). Boxes represent the interquartile range, horizontal lines indicate the median, whiskers represent the data range, and dots denote individual biological replicates. Differences among sites were assessed by ANOVA followed by Duncan’s multiple range test. Exact P values are shown, with significance thresholds of P < 0.01 and P < 0.001. JY, Tonglu Jianying Orchard; XL, Tonglu Xiaolu Orchard; ZS, Jiande Hongji Family Farm; WS, Jiande Strawberry Garden; CF, Changfeng Orchard; HS, Hangzhou Academy of Agricultural Sciences. B prefix, bacterial rhizosphere community; F prefix, fungal rhizosphere community; Shannon, Shannon diversity index; Simpson, Simpson diversity index; Chao1, Chao1 richness estimator; ACE, abundance-based coverage estimator.

#### 3.3.2 Spatial variation in bacterial community composition at the phylum and genus levels

Bacterial community composition exhibited marked spatial heterogeneity among sampling sites at both the phylum (**Figure 3A**) and genus (**Figure 3B**) levels. Four ubiquitous dominant phyla, *Pseudomonadota*, *Acidobacteriota*, *Chloroflexota*, and *Bacteroidota*, collectively accounted for more than 70% of all sequences. These phyla were most abundant at XL, JY, HS, and WS, respectively. *Actinobacteriota* and *Gemmatimonadota* were significantly enriched at CF compared with XL and the other sites. Five dominant genera contributed substantially to genus-level community differentiation, with most intersite differences reaching P < 0.01. Incertae_Sedis was most abundant at HS, whereas *Sphingomonas* and *Rhodanobacter* were enriched at JY. *Burkholderia*-*Caballeronia*-*Paraburkholderia* was particularly abundant at XL. *Nitrospira* and *Gemmatimonas* reached their highest relative abundances at ZS and CF, respectively. Overall, spatial variation in habitat conditions promoted the site-specific enrichment of bacterial taxa, resulting in marked differences in community composition among sampling locations.

**Figure 3:**
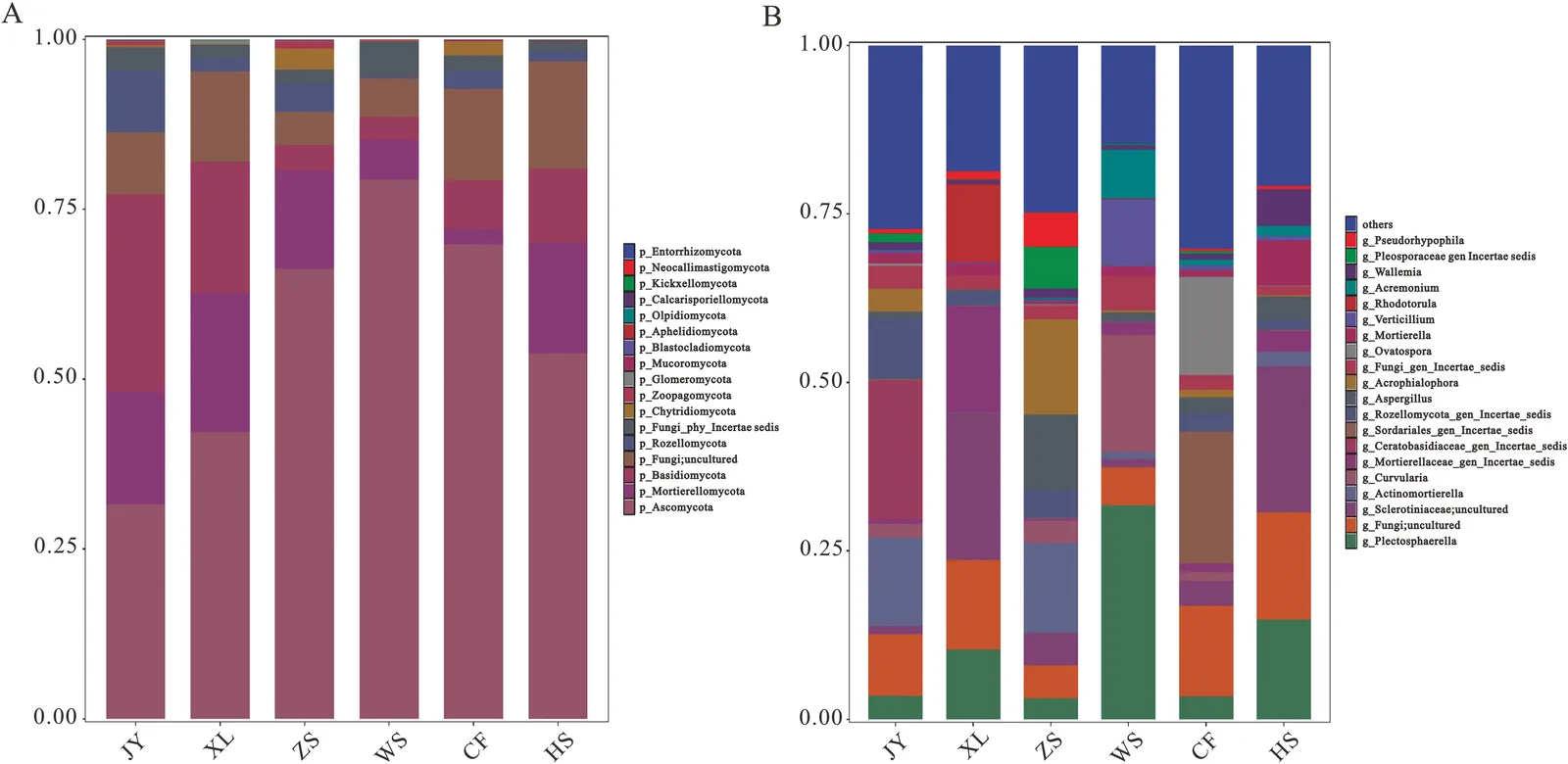
Relative composition of rhizosphere bacterial communities across six white strawberry cultivation sites. Bacterial community composition is presented at the **(A)** phylum and **(B)** genus levels. Each cultivation site comprised three independent biological replicates (n = 3), with each replicate consisting of a composite rhizosphere soil sample collected from five subsampling points. Bars show the mean relative abundance of bacterial taxa at each site, and taxa not displayed individually are grouped as “Others.” The figure provides a descriptive comparison of taxonomic composition, and no inferential statistical significance is indicated. The prefixes p_, g_, and f_ denote phylum, genus, and family, respectively. JY, Tonglu Jianying Orchard; XL, Tonglu Xiaolu Orchard; ZS, Jiande Hongji Family Farm; WS, Jiande Strawberry Garden; CF, Changfeng Orchard; HS, Hangzhou Academy of Agricultural Sciences. Taxonomic prefix definitions: k_, kingdom; p, phylum; f_, family; g_, genus.

#### 3.3.3 Spatial variation in fungal community composition at the phylum and genus levels

Fungal community composition at the phylum level differed significantly among sampling sites (**Figure 4A**). *Ascomycota*, *Mortierellomycota*, and *Basidiomycota* were the dominant core phyla, collectively accounting for more than 90% of the sequences across all samples. Clear site-specific dominance patterns were observed: *Ascomycota* predominated at WS, *Mortierellomycota* at XL, and *Basidiomycota* at JY. In addition, *Chytridiomycota* was significantly enriched at ZS, whereas *Rozellomycota* exhibited site-specific enrichment at JY. Site-level differences in fungal communities were primarily attributable to the differential distribution of dominant genera, and pairwise PERMANOVA revealed significant differences among sites (P < 0.01). The relative abundance of *Plectosphaerella* was highest at WS, whereas *Cephaliophora* was uniquely and strongly enriched at JY (**Figure 4B**). *Actinomortierella* was detected mainly at JY and ZS, while *Mortierella*, *Curvularia*, and *Acrophialophora* reached their highest relative abundances at HS, WS, and ZS, respectively.

**Figure 4:**
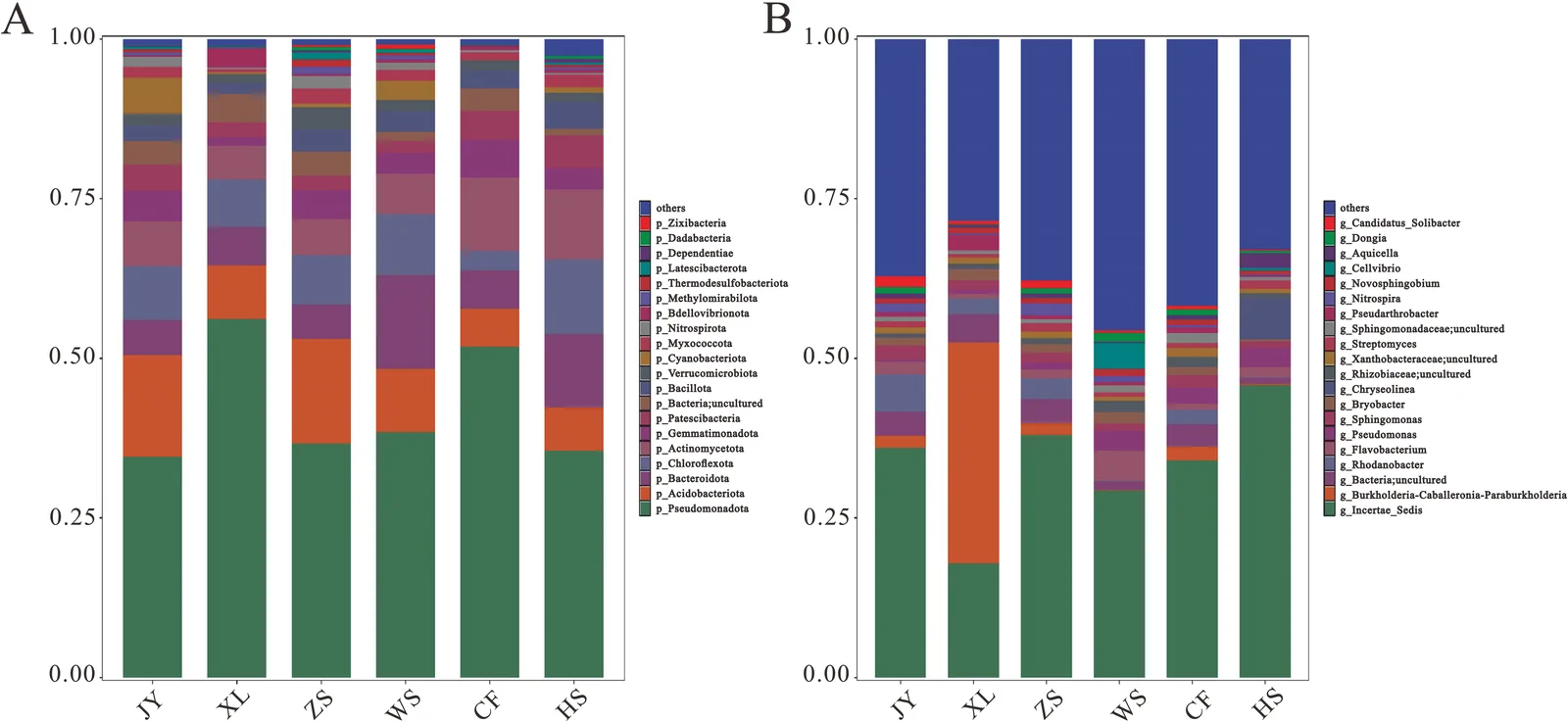
Relative composition of rhizosphere fungal communities across six white strawberry cultivation sites. Fungal community composition is presented at the **(A)** phylum and **(B)** genus levels. Each cultivation site comprised three independent biological replicates (n = 3). Taxa not displayed individually are grouped as “Others.” Differences in overall fungal community composition among sites were evaluated using pairwise permutational multivariate analysis of variance (PERMANOVA), with false discovery rate (FDR) correction applied for multiple comparisons. An adjusted P < 0.05 was considered statistically significant. The prefixes p_, g_, k_, and f_ denote phylum, genus, kingdom, and family, respectively. JY, Tonglu Jianying Orchard; XL, Tonglu Xiaolu Orchard; ZS, Jiande Hongji Family Farm; WS, Jiande Strawberry Garden; CF, Changfeng Orchard; HS, Zhijiang Base of the Hangzhou Academy of Agricultural Sciences.

### 3.4 Spatial variation of agronomic traits of strawberry plants

As shown in **Table 2**, the agronomic traits of ‘Cuiyu’ strawberry differed significantly among cultivation regions (P < 0.05). Among the vegetative growth traits, ZS had the greatest plant height, aboveground fresh weight, and belowground fresh weight (32.00 ± 6.71 g). In particular, belowground fresh weight at ZS was significantly greater than that at all other sites. By contrast, HS had the lowest plant height, which was significantly lower than those at the other sites. The maximum plant height and aboveground fresh weight recorded at ZS were 53.01% and 72.09% higher, respectively, than the corresponding minimum values at HS. Plants at XL had significantly larger crown diameters and longer roots, which may contribute to greater nutrient uptake and stress tolerance. Among the reproductive traits, HS produced the greatest numbers of pedicels and branches. The number of pedicels at HS was 230.71% higher than the minimum recorded at JY. CF had the greatest number of flowers and fruits, which was 89.81% higher than the lowest value observed at WS.

**Table 2:** Agronomic traits of ‘Cuiyu’ white strawberry plants across the six cultivation sites.

Sampling site	Plant height(cm)	Pedicel number(No. plant^-^¹)	Flower and fruit number(No. plant^-^¹)	Crown diameter(mm)	Branch number(No. plant^-^¹)	Aboveground fresh weight (g·plant^-^¹)	Underground fresh weight(g·plant^-^¹))	Root length (cm)
ZS	25.40 ± 2.57a	7.17 ± 2.14ab	49.67 ± 18.13ab	18.16 ± 1.95ab	2.83 ± 1.17ab	74.00 ± 17.41a	32.00 ± 6.71a	11.00 ± 1.85b
WS	22.80 ± 2.11ab	5.17 ± 2.79bc	36.00 ± 15.32b	17.00 ± 2.02abc	2.83 ± 1.33ab	58.18 ± 14.88abc	21.00 ± 4.25c	11.00 ± 1.54b
CF	21.60 ± 3.33b	8.00 ± 1.26ab	68.33 ± 12.42a	15.66 ± 1.54bc	1.83 ± 0.41b	49.00 ± 14.78bc	20.00 ± 0.36c	11.60 ± 2.86b
XL	20.60 ± 0.99b	6.00 ± 2.10ab	61.67 ± 20.66a	19.02 ± 0.59a	1.67 ± 0.51b	68.10 ± 21.52ab	23.00 ± 6.86c	16.30 ± 3.56a
JY	21.80 ± 1.92b	2.67 ± 0.52c	62.33 ± 7.78a	15.02 ± 2.54c	2.67 ± 0.82ab	56.00 ± 17.74abc	27.00 ± 10.88b	13.82 ± 2.15ab
HS	16.60 ± 3.15c	8.83 ± 2.64a	61.17 ± 16.85a	14.30 ± 2.33c	3.67 ± 1.21a	43.00 ± 15.65c	27.00 ± 14.44b	11.80 ± 1.90b

Values are presented as means ± SD (n = 6). Different superscript lowercase letters within the same column indicate significant differences among sampling sites according to ANOVA followed by Duncan’s multiple range test (P < 0.05). No. plant^-^¹, number per plant; ZS, Jiande Hongji Family Farm; WS, Jiande Strawberry Garden; CF, Changfeng Orchard; XL, Tonglu Xiaolu Orchard; JY, Tonglu Jianying Orchard; HS, Hangzhou Academy of Agricultural Sciences. No., Number; No. plant^-^¹, number per plant.

### 3.5 Spatial variations of leaf mineral elements and fruit quality of strawberry

As shown in **Figure 5**, the vegetative traits of ‘Cuiyu’ strawberry differed significantly among the six cultivation regions (P < 0.05). Plants at ZS had the greatest aboveground and belowground fresh weights, both of which were significantly higher than those recorded at the other sites. Leaf concentrations of macronutrients (N, P, and K), secondary nutrients (Ca and Mg), and micronutrients (Zn, Mn, and Fe) differed significantly among regions (**Figure 5**). ZS had the highest leaf total N and P concentrations, whereas CF had the lowest concentrations of both nutrients (**Figure 5A**). Leaf K concentration was highest at HS and lowest at XL. Although Ca and Mg showed less pronounced variation, significant regional differences were still detected. Among the micronutrients (**Figure 5B**), WS had the highest Zn concentration, XL had the highest Mn concentration, and CF had the highest Fe concentration. Zn and Mn concentrations varied substantially among regions. All fruit-quality indicators differed significantly among regions (**Figure 5C**). Fruits from WS had the highest individual fruit weight, total soluble solids (TSS), titratable acidity (TA), and soluble sugar content, whereas those from ZS had the lowest individual fruit weight. Fruit firmness at JY was significantly greater than that at WS and HS. Fruits from HS had the highest ascorbic acid concentration but the lowest TSS content, whereas those from XL had the lowest TA content. Additional Spearman correlation analyses revealed heterogeneous associations among soil nutrients, leaf mineral concentrations, vegetative traits, and individual fruit-quality parameters (**Supplementary Figure S1**). Soil nutrient variables showed coordinated variation, with strong positive correlations between SOM and HN (ρ = 0.83), RAP and pH (ρ = 0.80), and HN and CEC (ρ = 0.68). Several associations between soil nutrients and fruit-quality traits were also detected. AP was negatively correlated with WSF (ρ = −0.70), whereas CEC was positively correlated with HA (ρ = 0.69). Leaf mineral composition exhibited distinct element-specific relationships, including strong correlations between LeafP and LeafNC (ρ = 0.83), LeafP and LeafMg (ρ = −0.80), and LeafZn and both TA (ρ = 0.93) and SS (ρ = 0.78). Vegetative traits also showed variable associations with fruit quality. RT was positively correlated with HA (ρ = 0.60) but negatively correlated with SS (ρ = -0.65). Among the fruit-quality traits, TA was positively correlated with SS (ρ = 0.70), whereas several moderate negative associations with VC were observed. Overall, nutrient status and vegetative performance showed complex, trait-dependent relationships with fruit quality, with no universal negative trade-off among nutrient accumulation, vegetative growth, and fruit-quality attributes.

**Figure 5:**
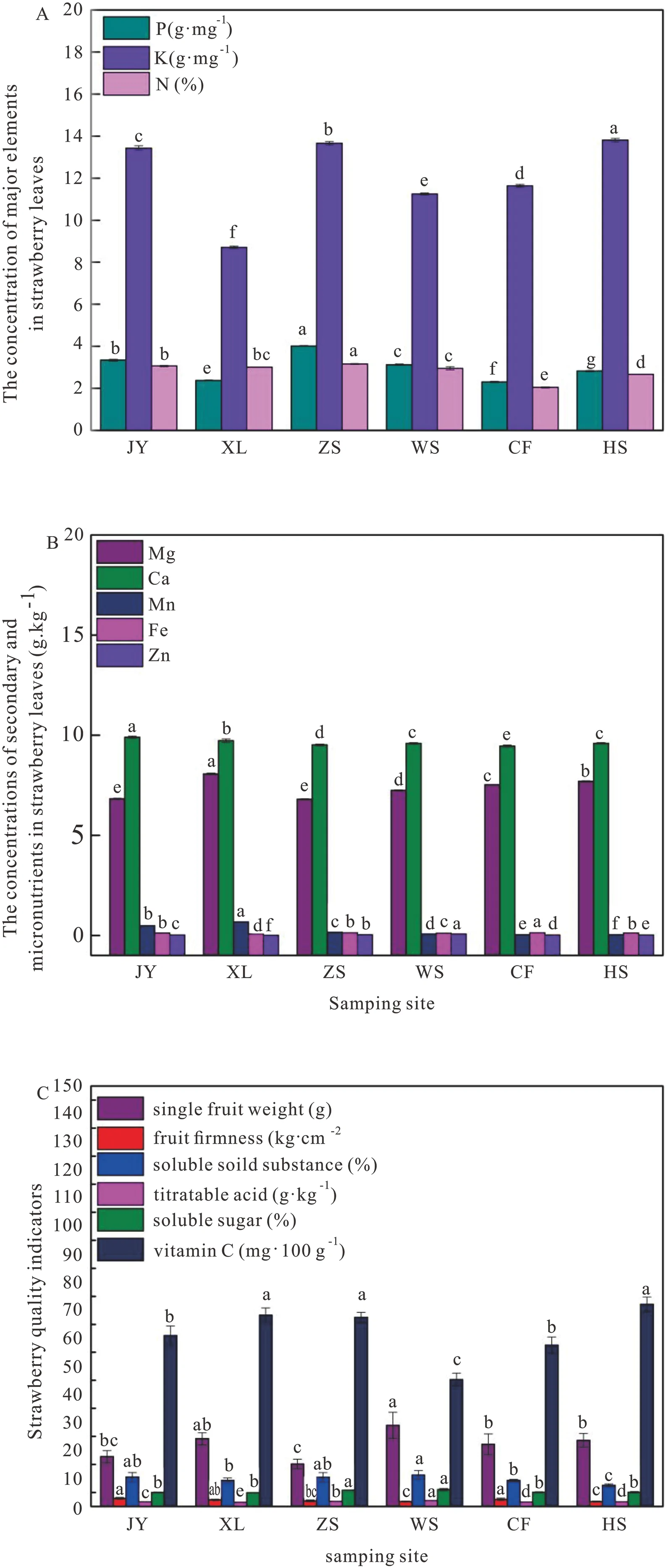
Spatial variation in leaf mineral elements and strawberry fruit quality across sampling sites. **(A)** Major elements. **(B)** Secondary and micronutrients. **(C)** Strawberry fruit-quality indicators. Leaf mineral abbreviations: N, total nitrogen; P, total phosphorus; K, total potassium; Ca, calcium; Mg, magnesium; Mn, manganese; Fe, iron; Zn, zinc. Fruit quality abbreviations: TSS, total soluble solid; TA, titratable acidity; Vc, vitamin **(C)** Values are presented as mean ± SD (n = 3). Different lowercase letters above the bars indicate significant differences among sampling sites, as determined by ANOVA followed by Duncan’s multiple-range test (P < 0.05). JY, Tonglu Jianying Orchard; XL, Tonglu Xiaolu Orchard; ZS, Jiande Hongji Family Farm; WS, Jiande Strawberry Garden; CF, Changfeng Orchard; HS, Hangzhou Academy of Agricultural Sciences.

### 3.6 Associations between soil factors and plant indicators revealed by Spearman correlation and Mantel tests

Spearman correlation analysis revealed coordinated variation among soil environmental variables (**Figure 6**). All pairwise correlations among bacterial α-diversity indices were positive and highly significant (P < 0.001). The correlation coefficients for BShannon-BSimpson, BShannon-BChao1, BShannon-BACE, BSimpson-BChao1, BSimpson-BACE, and BChao1-BACE were 0.926, 0.864, 0.812, 0.763, 0.730, and 0.971, respectively. Among the fungal diversity indices, FShannon was strongly correlated with FSimpson (r = 0.948, P < 0.001), while FChao1 was strongly correlated with FACE (r = 0.990, P < 0.001). Soil physicochemical properties and enzyme activities were also closely correlated. SOM was positively correlated with HN (r = 0.835, P < 0.001) and ACP (r = 0.723, P < 0.001), while HN was positively correlated with ACP (r = 0.889, P < 0.001). SC was positively correlated with both ACP (r = 0.773, P < 0.001) and urease activity (r = 0.767, P < 0.001). By contrast, CEC was negatively correlated with ACPT (r = -0.761, P < 0.001), and RAP was negatively correlated with CEC (r = -0.532, P < 0.05).

**Figure 6:**
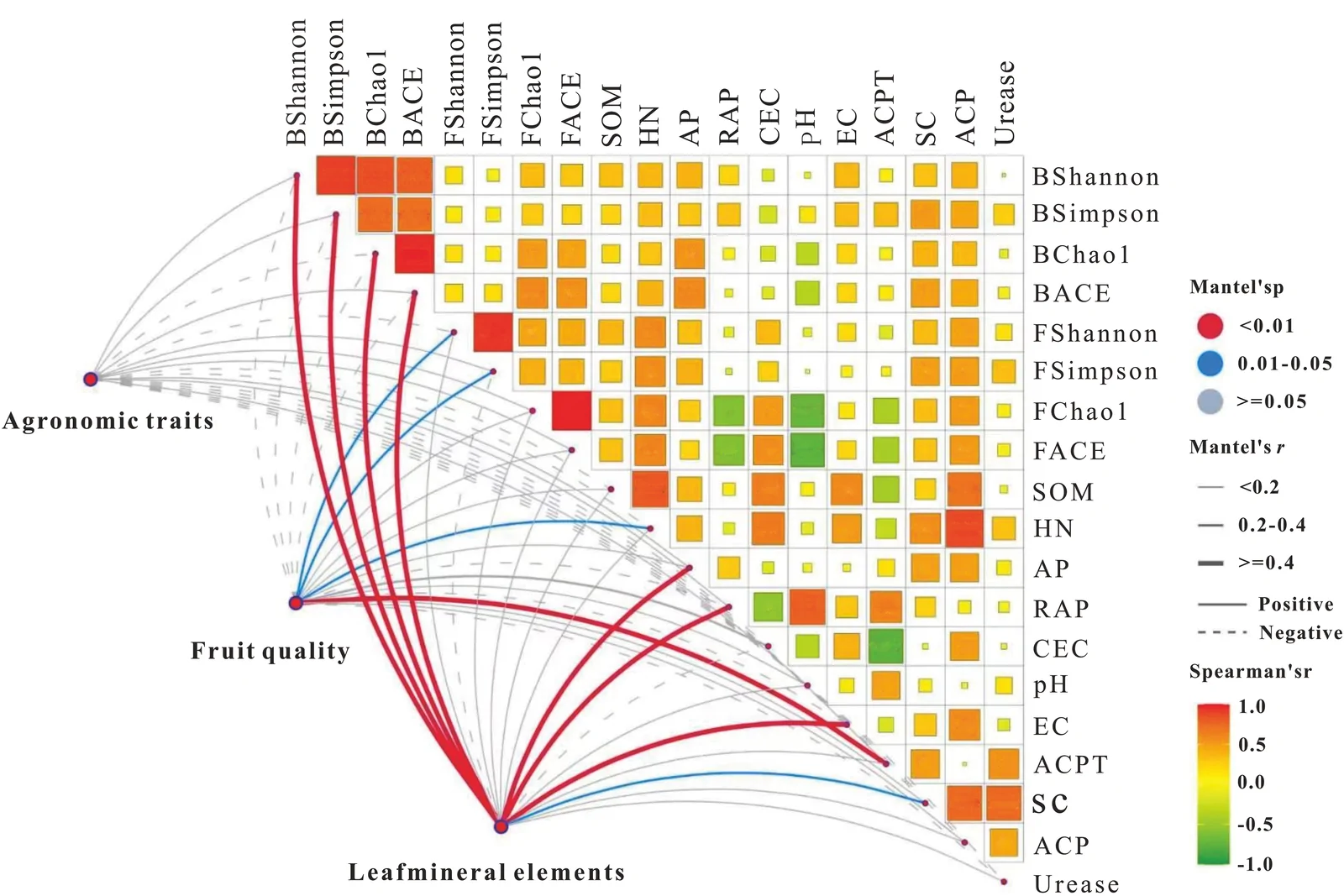
Mantel test analysis of relationships among soil physicochemical properties, rhizosphere microbiota, leaf mineral elements, and plant traits in strawberry cultivation. The upper panel shows a Spearman correlation matrix of soil physicochemical properties and microbial diversity indices. The color gradient represents Spearman correlation coefficients (r), with red and green indicating positive and negative correlations, respectively. Mantel tests were used to assess the relationships of soil and microbial characteristics with leaf mineral elements, agronomic traits, and fruit quality. Line color indicates the significance level of the Mantel test (P), line width represents the Mantel r statistic, and line type denotes positive or negative correlations. BShannon, BSimpson, BChao1, and BACE denote the bacterial Shannon, Simpson, Chao1, and ACE indices, respectively; FShannon, FSimpson, FChao1, and FACE denote the fungal Shannon, Simpson, Chao1, and ACE indices, respectively. SOM, soil organic matter; HN, hydrolyzable nitrogen; AP, available phosphorus; RAP, available potassium; CEC, cation exchange capacity; EC, electrical conductivity; ACPT, acid phosphatase activity; SC, sucrase activity; ACP, acid protease activity; Urease, urease activity.

Mantel tests detected no significant correlations between the agronomic-trait matrix and any soil factor (P ≥ 0.05, r = −0.162-0.162). However, the fruit-quality matrix was significantly correlated with FShannon (r = 0.348, P = 0.034), FSimpson (r = 0.371, P = 0.038), and HN (r = 0.260, P = 0.027), and was strongly correlated with ACPT (r = 0.465, P = 0.002). The leaf mineral-element matrix showed the strongest relationships with soil variables. It was significantly correlated with BShannon, BSimpson, BChao1, BACE, AP, RAP, and EC (r = 0.439–0.830, P < 0.01) and with SC (r = 0.223, P = 0.022). Overall, bacterial and fungal diversity exhibited distinct but complementary patterns of association with strawberry traits. Bacterial α-diversity was more strongly associated with leaf mineral profiles, whereas fungal Shannon and Simpson diversity were more closely associated with variation in fruit quality.

### 3.7 Analysis of the regulatory pathways between strawberry agronomic traits and fruit quality based on PLS-PM

The exploratory partial least squares path model had a descriptive goodness-of-fit value of 0.36 (GoF = 0.36; **Figure 7**). The model explained more variation in agronomic traits (R^2^ = 0.51) than in fruit quality (R^2^ = 0.28), indicating that it accounted more effectively for variation in strawberry vegetative and reproductive traits. Cultivation site and soil physicochemical properties showed positive modeled associations with soil enzyme activities, with path coefficients of 0.66 and 0.58, respectively. Soil enzyme activities and leaf mineral elements were also positively associated with agronomic traits, with corresponding path coefficients of 0.45 and 0.57. Total-effect analysis indicated that all upstream factors had negative overall associations with fruit quality. Leaf mineral elements made the largest negative contribution, while soil enzyme activities were also a major contributor. Overall, differences in soil environmental conditions were statistically associated with strawberry agronomic traits through modeled pathways involving soil enzyme activities and leaf mineral accumulation.

**Figure 7:**
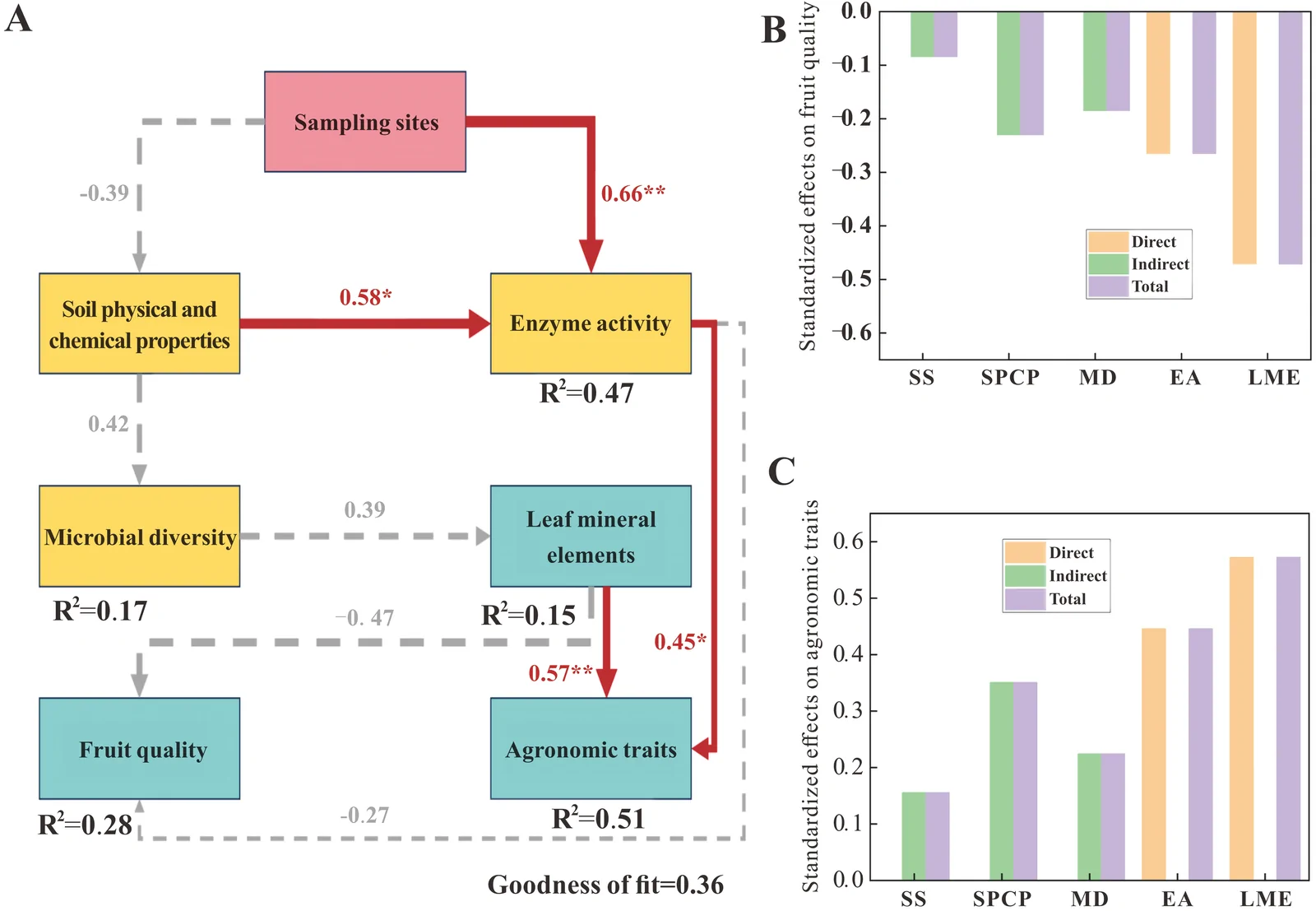
Partial least squares path modeling (PLS-PM) and standardized effect estimates. **(A)** PLS-PM showing the hypothesized relationships among sampling sites, soil physical and chemical properties, microbial diversity, enzyme activity, leaf mineral elements, agronomic traits and fruit quality. **(B)** Standardized direct, indirect and total effects of variables on fruit quality. **(C)** Standardized direct, indirect and total effects of variables on agronomic traits. Asterisks denote statistical significance: *P < 0.05, **P < 0.01. The numbers on the arrows are standardized path coefficients. R² indicates the proportion of variance explained. Goodness of fit represents the model fitness.

### 3.8 Associations of soil physicochemical properties with leaf properties accumulation revealed by RDA and exploratory PLS-PM

Using data on leaf mineral elements, soil physicochemical properties, soil enzyme activities, and microbial α-diversity from 18 samples, we examined the relationships between soil variables and mineral accumulation in strawberry leaves (**Figure 8**). RDA showed that all three groups of soil variables significantly explained variation in leaf mineral profiles. Permutation tests yielded adjusted proportions of explained variation of 89.5% for soil physicochemical properties, 73.3% for enzyme activities, and 71.1% for microbial α-diversity (**Figure 8A**). These findings indicated that leaf mineral accumulation was jointly associated with soil nutrient status, enzyme activity, and microbial diversity rather than with a single group of factors. Separate RDA models identified BACE, BChao1, AP, BSimpson, BShannon, RAP, pH, EC, SC, and ACPT as the dominant predictors. BACE and BChao1 made the largest contributions, indicating that bacterial richness was the principal microbial correlate of leaf mineral composition (**Figure 8B**). AP, soil pH, and EC were also strongly associated with mineral uptake and accumulation. The first two RDA axes collectively explained 74.9% of the total variation in leaf mineral profiles (**Figure 8C**). The small angles between the vectors representing soil variables and those representing P, K, Zn, and Fe indicated positive associations. By contrast, the larger angles with Ca, Mn, and Mg indicated weaker relationships. Leaf mineral profiles at CF, HS, and ZS were primarily associated with soil phosphorus, salinity, and bacterial diversity, whereas the profile at XL was less constrained by the measured soil variables.

**Figure 8:**
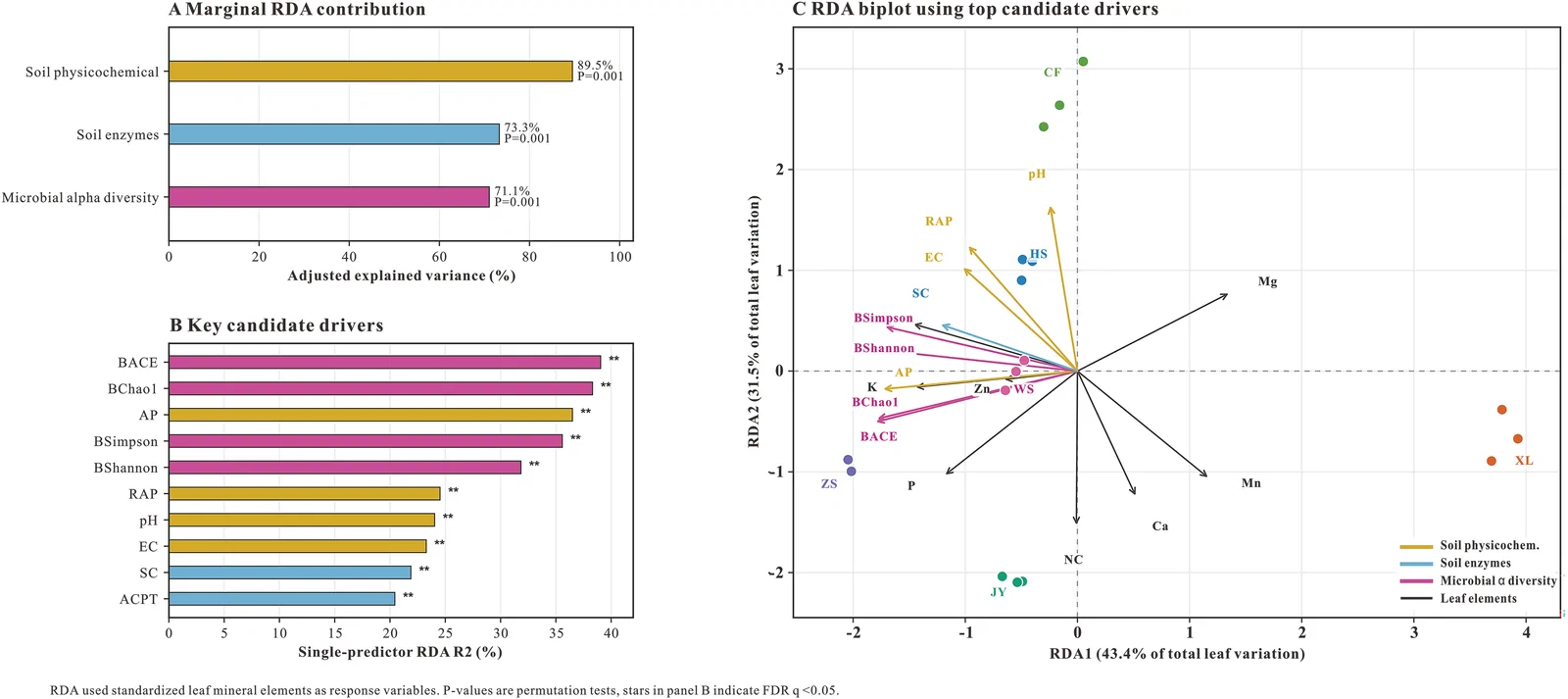
RDA of the relationships among soil physicochemical properties, microbial diversity, and leaf mineral element profiles across strawberry cultivation sites. **(A)** Marginal RDA showing the individual contributions of major predictor groups, including soil physicochemical properties, soil enzyme activities, and microbial alpha diversity, to variation in standardized leaf mineral element profiles. Values represent adjusted explained variance, and statistical significance was assessed using permutation tests. **(B)** Single-predictor RDA identifying candidate drivers of variation in leaf mineral element profiles. Bars represent the variance explained (R²) by individual predictors, and asterisks indicate significant contributions after FDR correction (q < 0.05). **(C)** RDA biplot illustrating the relationships among sampling sites, environmental variables, microbial diversity indices, and leaf mineral elements. Site scores are shown as colored points, whereas arrows represent explanatory and response variables. Arrow length reflects the strength of the contribution, and the angle between vectors reflects the direction and magnitude of their correlations. RDA was performed using standardized leaf mineral element concentrations as response variables, with soil physicochemical properties, soil enzyme activities, and microbial alpha-diversity indices included as explanatory variables. Statistical significance was evaluated using permutation tests. Site abbreviations: JY, Tonglu Jianying Orchard; XL, Tonglu Xiaolu Orchard; ZS, Jiande Hongji Family Farm; WS, Jiande Strawberry Garden; CF, Changfeng Orchard; HS, Hangzhou Academy of Agricultural Sciences Base. Variable abbreviations: SOM, soil organic matter; HN, hydrolyzable nitrogen; AP, available phosphorus; RAP, available potassium; CEC, cation exchange capacity; EC, electrical conductivity; ACPT, acid phosphatase activity; SC, sucrase activity; ACP, acid protease activity; Urease, urease activity; BShannon, bacterial Shannon index; BSimpson, bacterial Simpson index; BChao1, bacterial Chao1 index; BACE, bacterial ACE index; FShannon, fungal Shannon index; FSimpson, fungal Simpson index; FChao1, fungal Chao1 index; FACE, fungal ACE index. RDA, redundancy analysis; VIF, variance inflation factor; FDR, false discovery rate.

Mediation-based PLS-PM was used to estimate the modeled pathways linking soil physicochemical properties to leaf mineral accumulation. Soil physicochemical properties were positively associated with enzyme activities (path coefficient = 0.46) and microbial α-diversity (path coefficient = 0.47; **Figure 9**). Microbial α-diversity showed a strong positive association with leaf mineral status (path coefficient = 0.85), whereas enzyme activity had only a weak association. Effect decomposition identified positive microbe-mediated indirect effects but negligible enzyme-mediated effects. Overall, soil physicochemical properties were associated with leaf mineral accumulation primarily through changes in microbial α-diversity, with limited mediation by soil enzymes.

**Figure 9:**
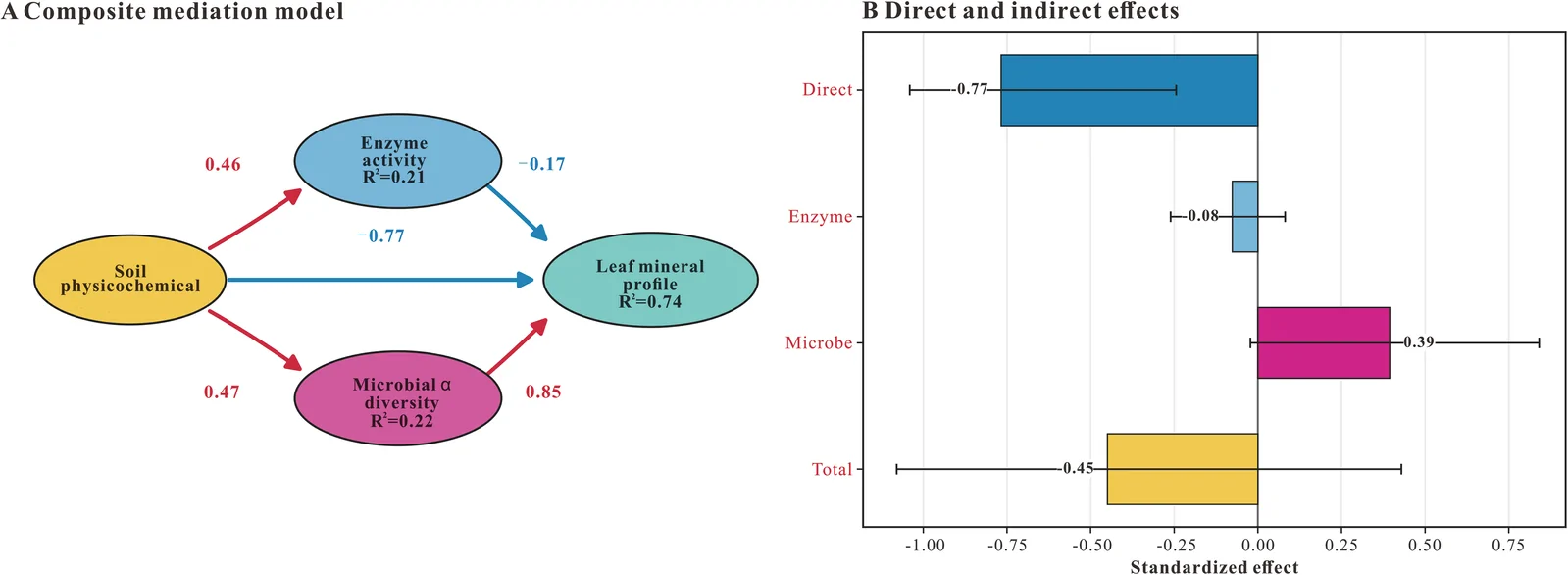
PLS-PM of the direct and indirect associations among soil physicochemical properties, microbial diversity, enzyme activities, and leaf mineral profiles in strawberry cultivation. **(A)** Conceptual PLS-PM model illustrating the hierarchical relationships among soil physicochemical properties, soil enzyme activities, microbial α-diversity, and leaf mineral profiles. Arrows denote hypothesized pathways, and the values shown along the arrows are standardized path coefficients. Positive and negative coefficients indicate positive and negative associations, respectively. R² values within the latent variables indicate the proportion of variance explained for each endogenous variable. **(B)** Standardized direct, indirect, and total effects of soil physicochemical properties on leaf mineral profiles. Bars represent standardized effect sizes, and error bars indicate the range of standardized path coefficients obtained from the leave-one-site-out sensitivity analysis. Positive and negative values indicate positive and negative effects, respectively. The PLS-PM model included soil physicochemical properties, soil enzyme activities, and microbial α-diversity as explanatory latent variables, with standardized leaf mineral element profiles as the response variable. Model significance was assessed using bootstrap resampling (n = 5, 000). Soil physicochemical properties comprised SOM, hydrolyzable nitrogen, AP, available potassium, pH, electrical conductivity, and CEC. Microbial α-diversity comprised bacterial and fungal Shannon, Simpson, Chao1, and ACE indices, whereas soil enzyme activities comprised soil protease, sucrase, acid phosphatase, and urease activities. PLS-PM, Partial Least Squares Path Modeling; bootstrap, bootstrap resampling.

## 4 Discussion

Soil pH ranged from 4.41 to 5.54 across the sampling sites, indicating consistently acidic conditions. By contrast, EC and soil nutrient indicators, including SOM, AN, AP, and AK, varied more widely, demonstrating substantial physicochemical heterogeneity among cultivation regions. Soil pH is a major factor constraining microbial diversity (Rousk et al., 2010), while gradients in nutrient availability and EC further influence microbial community assembly. Consistent with the resource-based theory of microbial community assembly (Trivedi et al., 2020), copiotrophic microorganisms tend to dominate nutrient-rich soils, whereas oligotrophic populations are favored in nutrient-deficient or highly stressful environments. The dominant bacterial genera identified in this study may perform distinct biogeochemical functions in soil nitrogen and phosphorus cycling. The enrichment of *Nitrospira* was consistent with the nitrification potential previously reported for members of this genus; however, neither nitrification activity nor the relevant functional genes were directly measured (Daims et al., 2015). *Sphingomonas* and *Rhodanobacter* have been associated with organic matter decomposition and nitrogen cycling in other soil systems, but whether these nutrient-transformation functions were active in the strawberry rhizosphere could not be determined (Prakash et al., 2012). The greater abundance of *Gemmatimonas* at low-phosphorus sites may be associated with the phosphorus-limitation responses reported previously (Ma et al., 2023). Nevertheless, 16S rRNA amplicon sequencing provides taxonomic profiles and cannot directly verify the *in situ* nutrient-cycling functions of *Sphingomonas*, *Rhodanobacter*, *Gemmatimonas*, or other detected genera. Functional-gene prediction, metagenomic or metatranscriptomic sequencing, and controlled microbial inoculation experiments are required to validate the proposed mechanisms of micronutrient mobilization and nutrient transformation. Soil enzyme activities were closely correlated with SOM and soil nitrogen pools, suggesting strong relationships with substrate availability and nutrient-cycling processes. However, RDA and PLS-PM indicated that microbial α-diversity, particularly the ACE and Chao1 indices, was more strongly associated with leaf mineral concentrations than were soil enzyme activities. Among the microbial indicators examined, bacterial richness therefore showed the strongest statistical association with leaf mineral accumulation (Delgado-Baquerizo et al., 2018).

The strong association between bacterial α-diversity and leaf mineral concentrations suggests that microbial diversity may integrate multiple ecological functions that are not adequately represented by individual soil chemical properties or enzyme activities. Greater bacterial richness is generally associated with functional redundancy, resource-use complementarity, and metabolic versatility, which may enhance the stability and efficiency of nutrient transformations across heterogeneous soil environments. Although these mechanisms were not directly quantified, our findings are consistent with the hypothesis that microbial diversity contributes to soil ecosystem multifunctionality rather than merely reflecting changes in taxonomic composition (Wagg et al., 2014; Delgado-Baquerizo et al., 2016). In agreement with studies emphasizing the importance of microbiome diversity in soil–plant nutrient interactions (Trivedi et al., 2020), bacterial α-diversity was a more informative predictor of leaf mineral status than fungal diversity or individual soil enzyme activities in the present acidic agricultural-soil dataset.

Fungi communities of fungal also played a role in distinguishing between the regions, but the associations were different from those of the bacterial communities. Estimates of the Chao1 and ACE diversity indices for the fungi differed significantly between the sites, while the Shannon and Simpson indices did not differ; hence, relatively constant diversity and evenness of the fungi can be assumed. At the same time, Mantel tests showed significant correlations between the diversity of fungi (Shannon and Simpson indices) and the variation in fruit quality.

Such results do not imply the unimportant function of fungi in strawberry rhizosphere functioning. Fungi represent important elements of soil ecosystems and are involved in nutrient cycling, organic matter decomposition, and interactions between plants and microorganisms. Nevertheless, no direct assessment of functional properties of fungi and their symbiosis was conducted and requires further research. Thus, bacteria and fungi are two parts of the rhizosphere microbiome that can affect plant performance through different ecological mechanisms.

The emphasis on bacterial diversity in this study reflects the observed association patterns rather than the exclusion of fungal contributions. Integrating functional profiling with targeted microbial experiments will be necessary to clarify these mechanisms.

The statistical analyses indicated a hierarchical pattern of associations among soil conditions, microbial attributes, leaf mineral status, and strawberry performance. Variation in soil physicochemical properties was associated with greater soil enzyme activity and bacterial α-diversity, and the model explained more variation in agronomic traits (R² = 0.51) than in fruit quality (R² = 0.28). PLS-PM further indicated that the relationships between soil physicochemical properties and plant performance were more strongly associated with bacterial α-diversity than with enzyme activity. These modeled pathways should, however, be interpreted as ecological correlations rather than experimentally validated causal relationships. Soil nutrient availability and salinity gradients were closely associated with changes in microbial diversity, which in turn showed the strongest modeled association with leaf mineral accumulation (path coefficient = 0.85). This finding is consistent with previous evidence that greater microbial diversity is positively associated with ecosystem multifunctionality (Delgado-Baquerizo et al., 2016). Soil enzyme activity had comparatively weak indirect effects, suggesting that community-level microbial diversity may be a more informative ecological indicator of plant nutrient acquisition than individual enzyme activities. Although the path model indicated an overall negative association with the composite fruit-quality variable, the additional correlation analyses revealed heterogeneous associations across individual quality traits. Therefore, this negative path should not be interpreted as evidence of a generalized growth–quality trade-off. Instead, the relationships among nutrient status, plant growth, and fruit quality are better understood as trait-specific and site-dependent (Lim et al., 2026; Stoeva et al., 2026).

To better understand the connection between nutrient levels and fruit quality, more Spearman correlations between soil nutrient levels, leaf mineral levels, vegetative properties, and fruit quality attributes were conducted (**Supplementary Figure S1**). It was revealed that there were trait-specific connections between nutrient levels, plant mineral levels, vegetative success, and fruit quality, but there was no general trade-off between nutrient levels and fruit quality variation.

Importantly, the proposed cascade should not be interpreted as experimental proof that increased bacterial richness enhances mineral uptake. Because this was an observational field study, the identified relationships represent ecological associations rather than causal effects. The consistent patterns observed across Mantel tests, ordination analyses, and PLS-PM support bacterial richness as an integrative ecological indicator, but the underlying mechanisms require validation through microbial manipulation, metagenomics/metatranscriptomics, and nutrient-tracing experiments.

The spatial co-occurrence of soil bacterial taxa and leaf trace-element concentrations represents statistical associations rather than direct evidence of microbial regulation. Amplicon sequencing alone cannot verify nutrient-mobilizing functions; therefore, these observations should be regarded as ecological hypotheses requiring validation by functional omics, microbial manipulation, and nutrient-flux measurements.

There are several limitations that need to be kept in mind while drawing inferences from the findings of this study. Since this study uses observations gathered in the field at six different cultivation sites, the associations found between microorganisms and plants must be understood to be just that, mere associations, and not necessarily an indication of any microbial control. This is due to the fact that no manipulation of microbes, inoculations, or validations were done to determine causality between certain types of microbial communities and the ability of plants to obtain nutrients. Establishing such causal relationships will require further studies integrating microbial transplantation, synthetic community inoculation, functional-omics analyses, and nutrient-tracing approaches.

Additional limitations involve the small sample size of independent observations included in the PLS-PM analysis (18 greenhouse-level observations from six cultivation regions). Despite being an appropriate technique for conducting exploratory research on complex ecological processes, the small sample size might reduce the statistical power of the technique, increase the uncertainty in parameter estimation, and make the results highly dependent on model specification. Therefore, the correlations found using PLS-PM must be considered exploratory and cannot be interpreted as causative relationships due to limitations of PLS-PM application in field-based ecological studies (Wang et al., 2023; Yang et al., 2024). Studies encompassing broader spatial gradients and independent validation datasets will be necessary to assess the robustness and generalizability of these relationships.

From an agronomic perspective, the present findings suggest that strawberry nutrient management should extend beyond simply increasing fertilizer inputs and instead account for soil nutrient balance, EC, and rhizosphere microbial diversity. Because greater nutrient availability and enhanced vegetative performance were not consistently accompanied by improved fruit quality, site-specific fertilization and salinity management may help limit excessive vegetative growth while maintaining desirable fruit-quality characteristics. Bacterial richness may also serve as a complementary biological indicator of soil fertility and plant mineral nutritional status. Accordingly, cultivation practices that preserve diverse native microbial communities, including balanced organic amendments and measures that limit excessive nutrient accumulation, may provide additional agronomic benefits. Moreover, bacterial taxa associated with Zn or Fe accumulation may represent promising candidates for future microbiome-based cultivation strategies. Their functional roles and agronomic effectiveness, however, remain to be established through controlled inoculation experiments and multi-season field trials.

Taken together, our findings support a conceptual cascade linking soil heterogeneity, microbial community assembly, and leaf mineral accumulation, whereby spatial variation in soil physicochemical properties is associated with corresponding shifts in rhizosphere microbial communities and leaf mineral status. Nevertheless, cultivation site represents an integrated environmental context rather than an experimentally controlled treatment. Unmeasured differences in greenhouse microclimate, management practices, and other site-specific factors may therefore have influenced both microbial community structure and plant performance. The observed relationships should consequently be interpreted as site-level ecological associations, with bacterial richness regarded as an integrative ecological indicator rather than an independently established causal driver. Future controlled studies incorporating microclimatic monitoring, irrigation-water characterization, detailed management records, functional-omics profiling, and direct measurements of nutrient fluxes will be needed to disentangle these interacting effects and critically evaluate the proposed framework.

Future studies incorporating broader spatial gradients, additional independent validation datasets, and multi-season controlled experiments will be required to further evaluate the robustness, generalizability, and applicability of the proposed soil–microbe–plant framework.

## 5 Conclusion

Across the six cultivation sites, soil physicochemical properties, rhizosphere microbiota, leaf mineral status, agronomic traits, and fruit quality varied markedly. Bacterial richness (ACE and Chao1) was most strongly associated with leaf mineral profiles, whereas fungal Shannon and Simpson diversity was more specifically associated with fruit-quality variation. RDA and exploratory PLS-PM identified bacterial α-diversity as the strongest microbial correlate of leaf mineral status, and soil-related variables explained more variation in agronomic traits than in fruit quality. These patterns represent ecological associations, not experimentally validated causal effects. Future studies should use controlled soil and microbial manipulations and targeted inoculation to validate candidate taxa, together with functional-omics and direct nutrient-flux or isotope-tracing measurements. Replication across regions, soil types, seasons, and years is needed to test robustness and separate microbial effects from site-specific environmental and management influences.

## Data Availability

The original contributions presented in the study are included in the article/**Supplementary Material**. Further inquiries can be directed to the corresponding authors.
