# Supplementary material for: Spatial associations among soil physicochemical properties, rhizosphere microbiota, leaf mineral status, agronomic traits, and fruit quality of white strawberry in acidic soils
Source: Front Plant Sci. 2026 Sep 21;17:1927019. doi: 10.3389/fpls.2026.1927019 (PMC13635311; doi:10.3389/fpls.2026.1927019)
Supplement: Supplementary file 1 [file SupplementaryFile1.docx]

**Figure S1.** Spearman correlation heatmaps showing associations of fruit-quality traits with soil physicochemical properties, leaf mineral concentrations, and agronomic traits.

(A) Correlations between soil physicochemical properties and fruit-quality traits.

(B) Correlations between leaf mineral concentrations and fruit-quality traits.

(C) Correlations between agronomic traits and fruit-quality traits.

Cell colors and numerical values represent Spearman correlation coefficients (ρ), with red and blue indicating positive and negative correlations, respectively. Asterisks indicate statistical significance after Benjamini–Hochberg FDR correction: *q < 0.05, **q < 0.01, and ***q < 0.001. Correlation analyses were performed using greenhouse-level biological replicates (n = 18).
