# Supplementary material for: Spatial associations among soil physicochemical properties, rhizosphere microbiota, leaf mineral status, agronomic traits, and fruit quality of white strawberry in acidic soils
Source: Front Plant Sci. 2026 Sep 21;17:1927019. doi: 10.3389/fpls.2026.1927019 (PMC13635311; doi:10.3389/fpls.2026.1927019)
Supplement: Supplementary file 3 [file DataSheet2.zip › Supplementary methods/Supplementary Methods 1.docx]

**Supplementary Methods**

*Spatial Associations Among Soil Physicochemical Properties, Rhizosphere Microbiota, Leaf Mineral Status, Agronomic Traits, and Fruit Quality of White Strawberry in Acidic Soils*

The following supplementary protocols provide the procedural detail underlying Sections 2.3, 2.4, 2.6, and 2.7 of the main manuscript. Measurements were conducted at the greenhouse-sample level, and analytical results were converted to the units reported in the manuscript. Unless otherwise stated, reagent blanks and calibration standards were processed in parallel with samples, and replicate measurements were used to check analytical precision.

**S1. Detailed determination of soil physicochemical properties**

Sample preparation. Composite bulk-soil samples were homogenized immediately after collection. The portion used for physicochemical analyses was air-dried at room temperature, gently disaggregated, and passed through a 2-mm sieve. Visible roots and plant residues were removed. A separate fresh-soil aliquot was used to determine gravimetric moisture content so that results requiring dry-mass correction could be expressed on an oven-dry soil-equivalent basis.

Soil pH and electrical conductivity. Soil pH was measured potentiometrically in a 1:2.5 (w/v) soil-to-deionized-water suspension. The suspension was mixed thoroughly and allowed to equilibrate before measurement with a calibrated glass-electrode pH meter. The meter was calibrated with at least two certified buffer solutions bracketing the sample pH range. EC was measured in an aqueous soil extract using a calibrated conductivity meter with temperature compensation, and values were reported as μS·cm⁻¹.

Soil organic matter (SOM). SOM was determined by potassium dichromate oxidation with external heating following Lu (2000). Air-dried soil was reacted with standardized K2Cr2O7 and concentrated H2SO4 under controlled heating. After cooling, residual dichromate was back-titrated with standardized FeSO4 solution using a suitable redox indicator. A reagent blank was carried through the complete procedure. Organic C was calculated from blank-corrected dichromate consumption and converted to SOM using the conventional organic C-to-organic matter conversion factor. Results were expressed as g·kg⁻¹ dry soil.

Alkaline hydrolyzable nitrogen (HN). HN was determined by alkaline hydrolysis-diffusion. A known mass of air-dried soil was exposed to standardized NaOH in a sealed diffusion unit, and the NH3 released during incubation was trapped in boric acid indicator solution. The trapped NH3 was quantified by titration with standardized acid. A blank without soil was analyzed simultaneously. HN was calculated from the blank-corrected titration volume and expressed as mg N·kg⁻¹ dry soil.

Available phosphorus (AP). AP in these acidic soils was determined after acid-fluoride extraction. The clarified extract was reacted with an acidified ammonium molybdate-antimony reagent and reducing agent to form molybdenum blue. Absorbance was determined spectrophotometrically against a KH2PO4 calibration curve. Reagent blanks and calibration standards covering the sample concentration range were included in each batch. AP was expressed as mg P·kg⁻¹ dry soil.

Rapidly available potassium (RAP). Exchangeable/rapidly available K was extracted from air-dried soil with neutral ammonium acetate. After shaking and filtration, K in the extract was quantified by flame photometry using K standard solutions prepared in the same extraction matrix. Results were expressed as mg K·kg⁻¹ dry soil.

Exchangeable Ca and Mg and available Fe, Mn, and Zn. Exchangeable Ca and Mg were determined in the appropriate salt extract and quantified by atomic absorption spectrophotometry (AAS). Available Fe, Mn, and Zn were quantified in the corresponding soil extract by AAS. Element-specific hollow-cathode lamps and analytical wavelengths were used (Ca, 422.7 nm; Mg, 285.2 nm; Fe, 248.3 nm; Mn, 279.5 nm; Zn, 213.9 nm). Multi-point calibration curves were generated from certified elemental standards, and procedural blanks were included. Concentrations were corrected for dilution and sample mass and reported as mg·kg⁻¹ dry soil.

Quality control for soil physicochemical analyses. All glassware was acid-washed where trace-element determination was involved. Deionized water and analytical-grade reagents were used throughout. Instrument calibration was checked at the beginning and end of each analytical batch. Reagent blanks, duplicate/replicate determinations, and calibration verification standards were analyzed with each batch; samples were reanalyzed when replicate agreement or calibration stability was unsatisfactory.

Reference:

Lu, R. K. (2000). Methods of agricultural soil chemical analysis. Beijing: China Agricultural Science and Technology Press.

Lindsay, W. L., & Norvell, W. A. (1978). Development of DTPA soil test for zinc, iron, manganese and copper. Soil Science Society of America Journal, 42, 421–428. doi:10.2136/sssaj1978.03615995004200030009x.
