# Supplementary material for: Spatial associations among soil physicochemical properties, rhizosphere microbiota, leaf mineral status, agronomic traits, and fruit quality of white strawberry in acidic soils
Source: Front Plant Sci. 2026 Sep 21;17:1927019. doi: 10.3389/fpls.2026.1927019 (PMC13635311; doi:10.3389/fpls.2026.1927019)
Supplement: Supplementary file 3 [file DataSheet2.zip › Supplementary methods/Supplementary Methods 2.docx]

**Supplementary Methods**

*Spatial Associations Among Soil Physicochemical Properties, Rhizosphere Microbiota, Leaf Mineral Status, Agronomic Traits, and Fruit Quality of White Strawberry in Acidic Soils*

The following supplementary protocols provide the procedural detail underlying Sections 2.3, 2.4, 2.6, and 2.7 of the main manuscript. Measurements were conducted at the greenhouse-sample level, and analytical results were converted to the units reported in the manuscript. Unless otherwise stated, reagent blanks and calibration standards were processed in parallel with samples, and replicate measurements were used to check analytical precision.

**S2. Detailed determination of soil enzyme activities**

General preparation and dry-mass correction. Field-moist soil was passed through a 2-mm sieve, stored at 4 °C, and analyzed within 24 h. Air-drying and grinding were avoided. Each enzyme assay included a sample reaction, an appropriate blank in which substrate or soil was omitted/inactivated, and analytical replicates. Enzyme activity was calculated on an oven-dry soil-equivalent basis using the measured gravimetric moisture content.

Sucrase (acid invertase). Fresh soil was incubated with sucrose substrate in acidic phosphate buffer at 37 °C for 24 h. After incubation and clarification, an aliquot of the extract was reacted with 3,5-dinitrosalicylic acid (DNS) reagent. Color was developed by heating in a boiling-water bath, followed by cooling and dilution. Absorbance was measured spectrophotometrically in the visible range used for the DNS-glucose assay, and glucose standards were treated identically. Activity was expressed as mg glucose released·g⁻¹ dry soil·d⁻¹. The reducing-sugar color reaction followed Miller (1959).

Urease. Fresh soil was incubated with 10% (w/v) urea substrate in citrate buffer (pH 6.7) at 37 °C for 24 h. After incubation, the suspension was clarified. Ammonium released from urea hydrolysis was quantified by the phenol-sodium hypochlorite color reaction; absorbance was read at 578 nm against an NH4+-N calibration curve. Urease activity was calculated after blank correction and expressed as μg NH4+-N released·g⁻¹ dry soil·d⁻¹.

Acid phosphatase. Acid phosphatase was assayed using p-nitrophenyl phosphate as substrate following the principle of Tabatabai and Bremner (1969). Fresh soil was incubated with modified universal buffer at pH 6.5 and p-nitrophenyl phosphate at 37 °C for 1 h. The reaction was stopped by addition of CaCl2 and NaOH, and the suspension was filtered or centrifuged to obtain a clear supernatant. The p-nitrophenol released was quantified spectrophotometrically at approximately 410 nm using a p-nitrophenol standard curve. Activity was expressed as nmol p-nitrophenol released·g⁻¹ dry soil·h⁻¹.

Acid protease. Acid protease activity was determined using casein as the substrate and the Folin-Ciocalteu color reaction. Fresh soil was incubated with casein in an acidic buffer under controlled temperature and time. The reaction was terminated with trichloroacetic acid to precipitate unhydrolyzed protein. After filtration/centrifugation, the soluble aromatic amino acids in the supernatant were reacted with alkaline carbonate and Folin-Ciocalteu reagent. Absorbance was measured spectrophotometrically against a tyrosine calibration curve. Activity was expressed as μg tyrosine equivalents released·g⁻¹ dry soil·h⁻¹.

Calculation. For each colorimetric enzyme assay, activity was calculated as A = (Cs − Cb) × V × DF / (mdry × t), where Cs and Cb are the concentrations obtained from the sample and blank calibration curves, respectively; V is the final extract or reaction volume represented by the measured aliquot; DF is the dilution factor; mdry is the oven-dry-equivalent mass of soil; and t is incubation time. The numerator was expressed as glucose, NH4+-N, p-nitrophenol, or tyrosine equivalents according to the enzyme assayed.

Quality control. Substrate blanks and soil blanks were included to correct for non-enzymatic color development and native analyte background. Standard curves were prepared freshly for each assay series. Samples were analyzed in replicate under identical substrate concentration, buffer, incubation temperature, and incubation time. Absorbance values exceeding the calibration range were remeasured after appropriate dilution.

Reference:

AOAC INTERNATIONAL. (2023). Official Methods of Analysis of AOAC INTERNATIONAL (22nd ed.). AOAC INTERNATIONAL, Rockville, MD. Methods 932.12 and 942.15.

Døving, A., & Måge, F. (2002). Methods of testing strawberry fruit firmness. Acta Agriculturae Scandinavica, Section B — Soil & Plant Science, 52(1), 43–51. doi:10.1080/090647102320260035.

Dubois, M., Gilles, K. A., Hamilton, J. K., Rebers, P. A., & Smith, F. (1956). Colorimetric method for determination of sugars and related substances. Analytical Chemistry, 28(3), 350–356. doi:10.1021/ac60111a017.

Wang, X. K. (2006). Principles and techniques of plant physiological and biochemical experiments (2nd ed.). Beijing: Higher Education Press.
