# Supplementary material for: Spatial associations among soil physicochemical properties, rhizosphere microbiota, leaf mineral status, agronomic traits, and fruit quality of white strawberry in acidic soils
Source: Front Plant Sci. 2026 Sep 21;17:1927019. doi: 10.3389/fpls.2026.1927019 (PMC13635311; doi:10.3389/fpls.2026.1927019)
Supplement: Supplementary file 3 [file DataSheet2.zip › Supplementary methods/Supplementary Methods 3.docx]

**Supplementary Methods**

*Spatial Associations Among Soil Physicochemical Properties, Rhizosphere Microbiota, Leaf Mineral Status, Agronomic Traits, and Fruit Quality of White Strawberry in Acidic Soils*

The following supplementary protocols provide the procedural detail underlying Sections 2.3, 2.4, 2.6, and 2.7 of the main manuscript. Measurements were conducted at the greenhouse-sample level, and analytical results were converted to the units reported in the manuscript. Unless otherwise stated, reagent blanks and calibration standards were processed in parallel with samples, and replicate measurements were used to check analytical precision.

**S3. Microbial DNA extraction, amplicon sequencing, and bioinformatic analysis**

DNA extraction and quality assessment. Total microbial DNA was extracted from 0.50 g homogenized rhizosphere soil using the CretMag™ Power Soil DNA Kit according to the manufacturer’s protocol. DNA integrity was evaluated by 1% agarose gel electrophoresis, and DNA concentration/purity were measured with a NanoDrop 2000 spectrophotometer. The overall amplicon-sequencing design followed established high-throughput marker-gene sequencing principles (Caporaso et al., 2012).

PCR amplification. The bacterial V3-V4 region was amplified with 341F/806R using the primer sequences reported in the main manuscript. The fungal ITS1 region was amplified with ITS1F and ITS2, whose source references are Gardes and Bruns (1993) and White et al. (1990), respectively. Reaction composition, thermal cycling conditions, triplicate amplification, pooling, purification, and DNBSEQ-G99 PE300 sequencing are reported in Section 2.5 of the main manuscript.

Read preprocessing and chimera control. Raw reads were quality-filtered with Trimmomatic (Bolger et al., 2014), merged with FLASH (Magoč & Salzberg, 2011) and PEAR (Zhang et al., 2014), and screened for chimeras with UCHIME (Edgar et al., 2011) implemented in VSEARCH (Rognes et al., 2016). The exact quality thresholds used in this study are provided in Section 2.5 of the main manuscript.

OTU clustering and taxonomic assignment. Sequence processing was conducted in QIIME 2 (Bolyen et al., 2019) and VSEARCH (Rognes et al., 2016). OTUs were clustered at 97% similarity using UPARSE (Edgar, 2013). Bacterial taxonomy was assigned against SILVA release 132 (Quast et al., 2013), and fungal taxonomy against UNITE release 7.2 (Nilsson et al., 2019).

Diversity normalization and quality control. OTU tables were rarefied only for α-diversity calculations following Weiss et al. (2017), whereas β-diversity analyses used unrarefied OTU tables. Sequencing depth adequacy was evaluated using rarefaction curves and Good’s coverage. The raw sequencing dataset accession

Reference:

Bolger, A. M., Lohse, M., & Usadel, B. (2014). Trimmomatic: a flexible trimmer for Illumina sequence data. Bioinformatics, 30(15), 2114–2120. doi:10.1093/bioinformatics/btu170.

Caporaso, J. G., Lauber, C. L., Walters, W. A., Berg-Lyons, D., Huntley, J., Fierer, N., et al. (2012). Ultra-high-throughput microbial community analysis on the Illumina HiSeq and MiSeq platforms. ISME Journal, 6, 1621–1624. doi:10.1038/ismej.2012.8.

Gardes, M., & Bruns, T. D. (1993). ITS primers with enhanced specificity for basidiomycetes—application to the identification of mycorrhizae and rusts. Molecular Ecology, 2(2), 113–118. doi:10.1111/j.1365-294X.1993.tb00005.x.

Nilsson, R. H., Larsson, K.-H., Taylor, A. F. S., Bengtsson-Palme, J., Jeppesen, T. S., Schigel, D., et al. (2019). The UNITE database for molecular identification of fungi: handling dark taxa and parallel taxonomic classifications. Nucleic Acids Research, 47(D1), D259–D264. doi:10.1093/nar/gky1022.

Weiss, S., Xu, Z. J., Peddada, S., Amir, A., Bittinger, K., Gonzalez, A., et al. (2017). Normalization and microbial differential abundance strategies depend upon data characteristics. Microbiome, 5(1), 27. doi:10.1186/s40168-017-0237-y.

White, T. J., Bruns, T., Lee, S., & Taylor, J. (1990). Amplification and direct sequencing of fungal ribosomal RNA genes for phylogenetics. In M. A. Innis, D. H. Gelfand, J. J. Sninsky, & T. J. White (Eds.), PCR Protocols: A Guide to Methods and Applications (pp. 315–322). Academic Press.

Quast, C., Pruesse, E., Yilmaz, P., Gerken, J., Schweer, T., Yarza, P., et al. (2013). The SILVA ribosomal RNA gene database project: improved data processing and web-based tools. Nucleic Acids Research, 41(D1), D590–D596. doi:10.1093/nar/gks1219.
