# Supplementary material for: Spatial associations among soil physicochemical properties, rhizosphere microbiota, leaf mineral status, agronomic traits, and fruit quality of white strawberry in acidic soils
Source: Front Plant Sci. 2026 Sep 21;17:1927019. doi: 10.3389/fpls.2026.1927019 (PMC13635311; doi:10.3389/fpls.2026.1927019)
Supplement: Supplementary file 3 [file DataSheet2.zip › Supplementary methods/Supplementary Methods 4.docx]

**Supplementary Methods**

*Spatial Associations Among Soil Physicochemical Properties, Rhizosphere Microbiota, Leaf Mineral Status, Agronomic Traits, and Fruit Quality of White Strawberry in Acidic Soils*

The following supplementary protocols provide the procedural detail underlying Sections 2.3, 2.4, 2.6, and 2.7 of the main manuscript. Measurements were conducted at the greenhouse-sample level, and analytical results were converted to the units reported in the manuscript. Unless otherwise stated, reagent blanks and calibration standards were processed in parallel with samples, and replicate measurements were used to check analytical precision.

**S4. Detailed determination of strawberry fruit-quality parameters**

Fruit sampling and homogenization. Commercially ripe fruits from the six subsample plants were collected synchronously within each greenhouse. Fruits were free of visible disease or mechanical damage. After individual fruit weight and firmness measurements, representative fruit tissue was pooled within each greenhouse and homogenized. Fresh homogenate or clarified juice was used immediately for TSS, soluble sugar, vitamin C, and titratable-acidity measurements to minimize compositional change during storage.

Individual fruit weight and firmness. Individual fruit weight was determined gravimetrically with a calibrated electronic balance following the standard plant physiological and biochemical measurement procedures summarized by Wang (2006). Firmness was measured at the fruit equator using a digital texture analyzer following established strawberry firmness-testing principles (Døving & Måge, 2002). The same probe geometry, penetration/deformation settings, and test position were used for all fruits within an analytical series.

Total soluble solids (TSS). TSS was determined by refractometry following AOAC Official Method 932.12 (AOAC INTERNATIONAL, 2023). Clarified strawberry juice was placed on a calibrated handheld refractometer at room temperature; the instrument was zeroed with deionized water, and TSS was reported as °Brix.

Soluble sugar. Soluble sugars were quantified by the phenol-sulfuric acid method of Dubois et al. (1956). An appropriately diluted fruit extract was reacted with phenol and concentrated H2SO4, and absorbance was measured after color development against a glucose calibration curve. Results were expressed on a fresh-mass basis.

Vitamin C (Vc). Vitamin C was determined by iodometric titration following the plant biochemical analytical procedure summarized by Wang (2006). Fresh fruit homogenate was extracted under acidic conditions, clarified, and titrated with standardized iodine solution using starch near the endpoint. Vc concentration was calculated from iodine consumption and expressed on a fresh-mass basis.

Titratable acidity (TA). TA was determined by standardized NaOH titration following AOAC Official Method 942.15 (AOAC INTERNATIONAL, 2023). Clarified fruit juice was titrated to the conventional endpoint near pH 8.1, and TA was expressed as citric-acid equivalents.

Quality control for fruit-quality analyses. Refractometers, balances, and texture analyzers were checked/calibrated before measurements. Colorimetric assays included reagent blanks and freshly prepared calibration standards. Titrants were standardized before use, and titration blanks were included. Replicate measurements were performed on the same greenhouse composite, and samples outside the validated analytical range were diluted and reanalyzed.

Reference:

Jesmin, T., Margenot, A. J., & Mulvaney, R. L. (2022). A comprehensive method for casein-based assay of soil protease activity. Communications in Soil Science and Plant Analysis, 53(4), 507–520. doi:10.1080/00103624.2021.2017954.

Tabatabai, M. A., & Bremner, J. M. (1969). Use of p-nitrophenyl phosphate for assay of soil phosphatase activity. Soil Biology and Biochemistry, 1, 301–307. doi:10.1016/0038-0717(69)90012-1.

Tabatabai, M. A., & Bremner, J. M. (1972). Assay of urease activity in soils. Soil Biology and Biochemistry, 4, 479–487. doi:10.1016/0038-0717(72)90064-8.

Miller, G. L. (1959). Use of dinitrosalicylic acid reagent for determination of reducing sugar. Analytical Chemistry, 31, 426–428. doi:10.1021/ac60147a030.

Murphy, J., & Riley, J. P. (1962). A modified single solution method for the determination of phosphate in natural waters. Analytica Chimica Acta, 27, 31–36. doi:10.1016/S0003-2670(00)88444-5.
