# Supplementary material for: Spatial associations among soil physicochemical properties, rhizosphere microbiota, leaf mineral status, agronomic traits, and fruit quality of white strawberry in acidic soils
Source: Front Plant Sci. 2026 Sep 21;17:1927019. doi: 10.3389/fpls.2026.1927019 (PMC13635311; doi:10.3389/fpls.2026.1927019)
Supplement: Supplementary file 3 [file DataSheet2.zip › Supplementary methods/Supplementary Methods 5.docx]

**Supplementary Methods**

*Spatial Associations Among Soil Physicochemical Properties, Rhizosphere Microbiota, Leaf Mineral Status, Agronomic Traits, and Fruit Quality of White Strawberry in Acidic Soils*

The following supplementary protocols provide the procedural detail underlying Sections 2.3, 2.4, 2.6, and 2.7 of the main manuscript. Measurements were conducted at the greenhouse-sample level, and analytical results were converted to the units reported in the manuscript. Unless otherwise stated, reagent blanks and calibration standards were processed in parallel with samples, and replicate measurements were used to check analytical precision.

**S5. Agronomic traits and leaf mineral-element analysis**

Agronomic traits. Agronomic measurements followed standard plant physiological, morphometric, and gravimetric procedures summarized by Wang (2006). Six representative plants within each greenhouse were measured as subsamples at the same sampling stage. Plant height and root length were measured with a graduated ruler; crown diameter was measured with a digital vernier caliper; pedicel number, branch number, and flower and fruit number were recorded by direct counting per plant; and aboveground and belowground fresh weights were determined immediately using a calibrated analytical balance after removal of adhering debris with deionized water. The same operator and measurement criteria were used across cultivation sites to minimize procedural variation.

Leaf sample preparation. Fully expanded healthy leaves collected from the six subsample plants were pooled within each greenhouse. Leaves were rinsed with deionized water, dried at 65 °C to constant mass, ground to a homogeneous fine powder, and stored in clean sealed containers until digestion. Separate analytical aliquots were used for macronutrient and metal determinations when required by the analytical platform.

Nitrogen, phosphorus, and potassium. Leaf N was measured after H2SO4-H2O2 wet digestion by continuous-flow analysis following Umar et al. (2023) and the Kjeldahl digestion principle (Kjeldahl, 1883). Leaf P was determined by molybdenum-antimony colorimetry (Murphy & Riley, 1962), and K by flame photometry following the procedures summarized by Lu (2000).

Calcium, magnesium, iron, manganese, and zinc. Dried leaf material was digested to obtain a clear mineral solution, and Ca, Mg, Fe, Mn, and Zn were quantified by atomic absorption spectrophotometry following Lu (2000). Calibration curves, digestion blanks, and verification standards were included in each analytical batch.

Quality control for leaf mineral analysis. Digestion blanks and calibration verification standards were included in each batch. Calibration curves bracketed the sample concentration range. Samples with concentrations above the highest standard were diluted and reanalyzed. Duplicate digestions or replicate instrumental readings were used to verify analytical precision, and all concentrations were corrected for reagent blanks and dilution.

References

Murphy, J., & Riley, J. P. (1962). A modified single solution method for the determination of phosphate in natural waters. Analytica Chimica Acta, 27, 31–36. doi:10.1016/S0003-2670(00)88444-5.

Lu, R. K. (2000). Methods of agricultural soil chemical analysis. Beijing: China Agricultural Science and Technology Press.

Umar, W. K., Arwendah, H., & Sustiyani, E. (2023). Nitrogen determination in oil-palm leaves using the San++ Scalar continuous-flow analyzer following sulfuric-acid wet digestion: testing and validation. Chimica et Natura Acta, 11(3), 125–135. doi:10.24198/cna.v11.n3.48210.

Wang, X. K. (2006). Principles and techniques of plant physiological and biochemical experiments (2nd ed.). Beijing: Higher Education Press.

Kjeldahl, J. Z. (1883). A new method for the determination of nitrogen in organic matter. Zeitschrift für Analytische Chemie, 22, 366–382. doi:10.1007/BF02514058.
